# Essential role of the Pax5 C-terminal domain in controlling B cell commitment and development

**DOI:** 10.1084/jem.20230260

**Published:** 2023-09-19

**Authors:** Sarah Gruenbacher, Markus Jaritz, Louisa Hill, Markus Schäfer, Meinrad Busslinger

**Affiliations:** 1 Research Institute of Molecular Pathology, Vienna BioCenter, Vienna, Austria; 2 Vienna BioCenter PhD Program, Doctoral School of the University of Vienna and Medical University of Vienna, Vienna, Austria

## Abstract

The B cell regulator Pax5 consists of multiple domains whose function we analyzed in vivo by deletion in *Pax5*. While B lymphopoiesis was minimally affected in mice with homozygous deletion of the octapeptide or partial homeodomain, both sequences were required for optimal B cell development. Deletion of the C-terminal regulatory domain 1 (CRD1) interfered with B cell development, while elimination of CRD2 modestly affected B-lymphopoiesis. Deletion of CRD1 and CRD2 arrested B cell development at an uncommitted pro-B cell stage. Most Pax5-regulated genes required CRD1 or both CRD1 and CRD2 for their activation or repression as these domains induced or eliminated open chromatin at Pax5-activated or Pax5-repressed genes, respectively. Co-immunoprecipitation experiments demonstrated that the activating function of CRD1 is mediated through interaction with the chromatin-remodeling BAF, H3K4-methylating Set1A-COMPASS, and H4K16-acetylating NSL complexes, while its repressing function depends on recruitment of the Sin3-HDAC and MiDAC complexes. These data provide novel molecular insight into how different Pax5 domains regulate gene expression to promote B cell commitment and development.

## Teaser

In vivo deletion in the mouse reveals an essential role of the Pax5 C-terminal domain in B cell commitment and development by activating or repressing most Pax5 target genes. This domain induces chromatin changes by interacting with chromatin-remodeling and histone-modifying complexes.

## Introduction

The transcription factor Pax5 is a major regulator of B lymphopoiesis and midbrain patterning. Within the hematopoietic system, Pax5 is specifically expressed only in the B-lymphoid lineage where it controls different aspects of B cell development and immunity. At the onset of B lymphopoiesis, Pax5 is essential for the commitment of lymphoid progenitors to the B cell lineage (Nutt et al., 1999). In pro-B cells, Pax5 promotes chromatin loop extrusion across the entire immunoglobulin heavy-chain (*Igh*) locus to facilitate the participation of all V_H_ genes in V_H_-DJ_H_ recombination, which generates a broad B cell antigen receptor (BCR) repertoire (Fuxa et al., 2004; Hill et al., 2020). Pax5 is furthermore required for the generation of all mature B cell types and thus for all B cell immune responses in part by controlling BCR signaling (Calderón et al., 2021; Horcher et al., 2001). Human *PAX5* also plays a key role in B cell acute lymphoblastic leukemia (B-ALL) as a haploinsufficient tumor suppressor gene (Gu et al., 2019; Mullighan et al., 2007) as well as a partner gene of different oncogenic *PAX5* translocations (Coyaud et al., 2010; Nebral et al., 2009). Recently, *PAX5* mutations have also been shown to cause autism spectrum disorder by affecting cerebellar morphogenesis and midbrain neurogenesis (Kaiser et al., 2022).

Pax5 acts both as a transcriptional repressor to suppress B-lineage-inappropriate genes (Delogu et al., 2006) as well as an activator to induce gene expression required for B cell development and function (Schebesta et al., 2007). Moreover, Pax5 is known to regulate distinct transcriptional programs in early and late B lymphopoiesis (Revilla-i-Domingo et al., 2012). It furthermore functions as an epigenetic regulator by recruiting histone-modifying complexes to its target genes, which can either induce accessible, active chromatin at Pax5-activated genes or eliminate open chromatin at Pax5-repressed genes (McManus et al., 2011; Revilla-i-Domingo et al., 2012).

**Figure 1.**
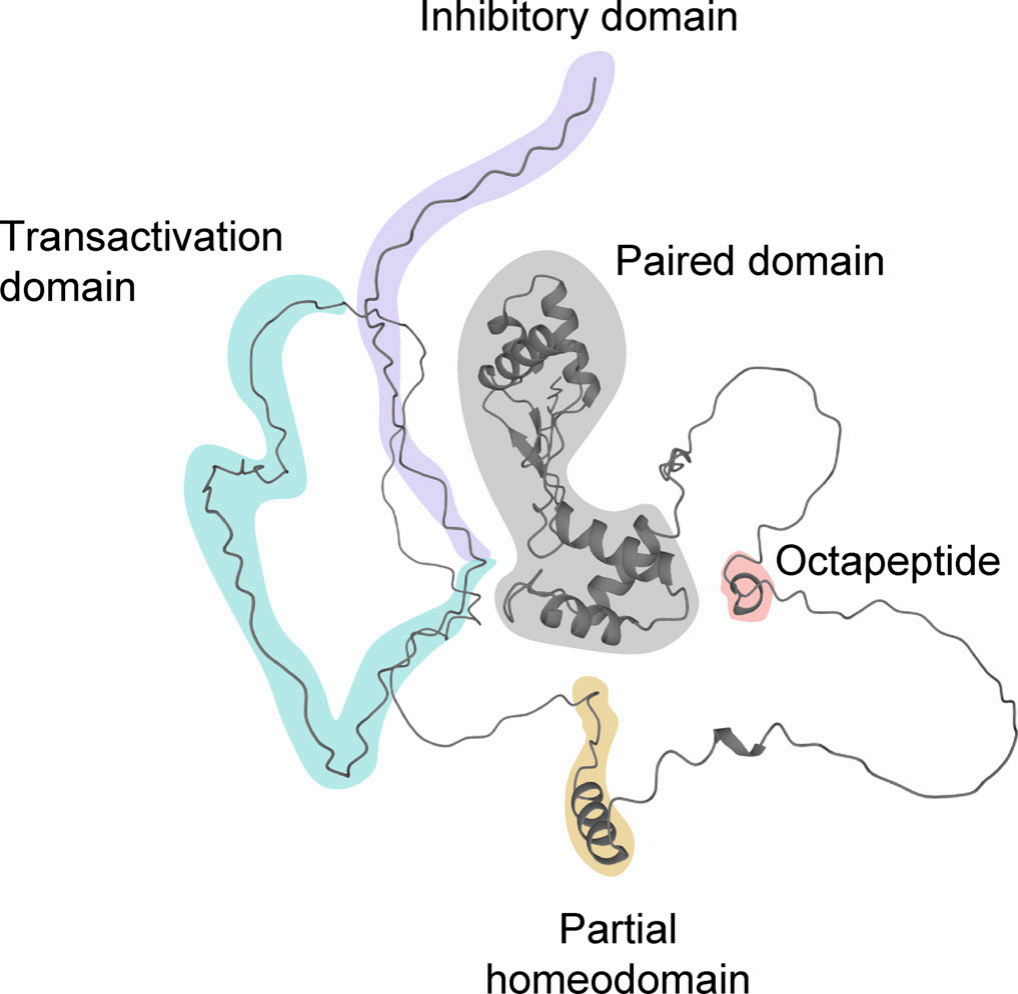
**Prediction of the Pax5 protein structure by AlphaFold.** The machine learning method AlphaFold (Jumper et al., 2021) correctly identified the structure of the bipartite paired domain of Pax5 that was previously defined by x-ray crystallography (Garvie et al., 2001). AlphaFold newly predicts α-helical structures for the OP and HD and furthermore indicates that the C-terminal transactivation and inhibitory domains of Pax5 (Dörfler and Busslinger, 1996) are unstructured. The C-terminal domains are referred to as CRD1 and CRD2 in this publication.

The Pax5 protein consists of several evolutionarily conserved domains (Bouchard et al., 2008). The DNA-binding function of Pax5 is encoded by the N-terminal paired domain (Czerny et al., 1993; Garvie et al., 2001). The conserved octapeptide motif (OP) of Pax5 is present in the central region of all vertebrate Pax proteins except in Pax4 and Pax6 (Bouchard et al., 2008) and is known to bind corepressors of the Groucho (Grg/Tle) protein family (Eberhard et al., 2000). While Pax3, Pax4, Pax6, and Pax7 contain a homeodomain with three α-helices as a second DNA-binding region in addition to the paired domain (Wilson et al., 1995), the subfamily of Pax2, Pax5, and Pax8 is characterized by the presence of a partial homeodomain (HD) sequence consisting of only the first α-helix (Bouchard et al., 2008). The HD of Pax5 is known to interact with the TATA-binding protein of the transcription initiation complex TFIID (Eberhard and Busslinger, 1999). The machine learning method AlphaFold (Jumper et al., 2021) correctly identifies the known structure of the Pax5 paired domain (Garvie et al., 2001) but also newly predicts α-helical structures for the OP and HD of Pax5 (Fig. 1). Interestingly, however, the C-terminal sequences of Pax5, which are highly conserved also in Pax2 and Pax8 (Dörfler and Busslinger, 1996), are predicted to be intrinsically disordered (Fig. 1). Our previous characterization of the C-terminal sequences of Pax5 by mutagenesis and transient transfection assay in established B cell lines identified a potent transactivation domain (TAD) and an adjacent inhibitory domain (ID; Dörfler and Busslinger, 1996). In contrast to these C-terminal sequences, the OP and HD of Pax5 have not yet been analyzed with regard to their gene-regulatory function. Moreover, although we have identified Pax5-activated and Pax5-repressed genes in early pro-B cells and mature follicular (FO) B cells (Revilla-i-Domingo et al., 2012), we still know little about how Pax5 regulates these genes in vivo. Here, we have studied the function of the different Pax5 domains in vivo by deletion in the endogenous *Pax5* gene, which provided novel mechanistic insight into the role of these domains in regulating gene expression and B cell development.

## Results

### The central domains of Pax5 contribute to optimal B cell development

**Figure 2.**
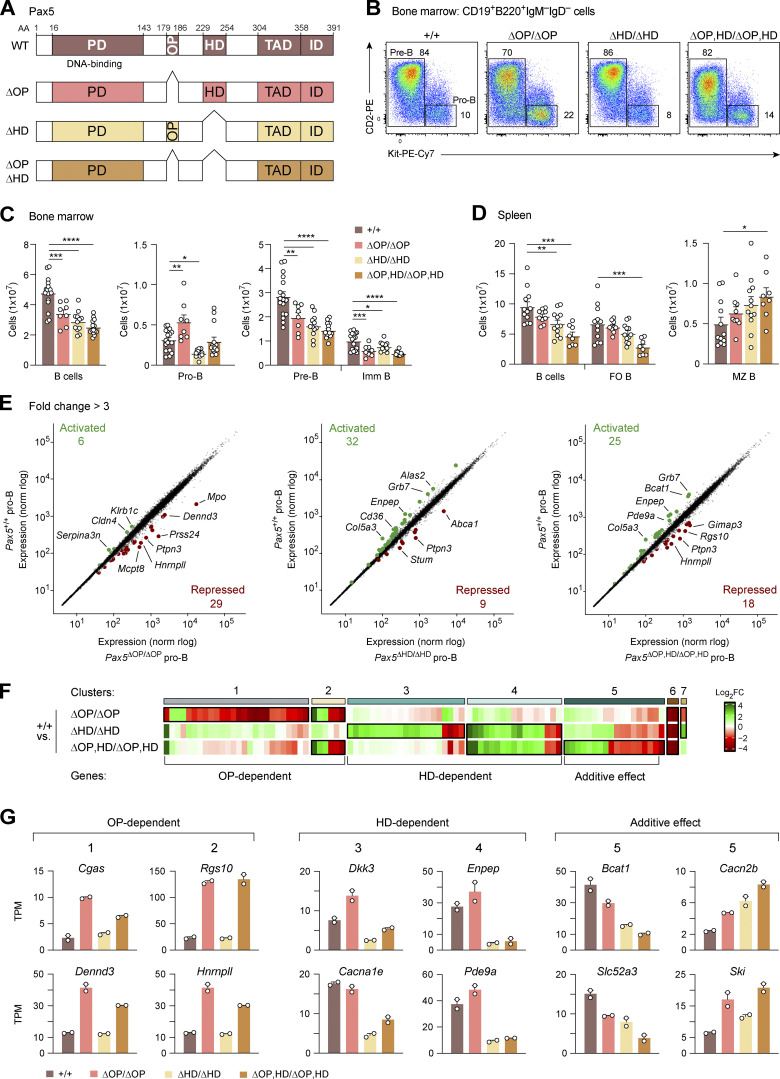
**Role of the central Pax5 domains in B cell development and gene regulation. (A)** Schematic diagram of the domain organization of Pax5 consisting of the paired domain (PD), OP, HD, TAD, and ID (Dörfler and Busslinger, 1996). The extent of sequence deletion present in the *Pax5*^∆OP^, *Pax5*^∆HD^, and *Pax5*^∆OP,HD^ alleles are indicated together with the respective amino acid positions. The deleted DNA sequences are shown in Fig. S1 A. WT, full-length Pax5 protein of the wild-type mouse. **(B)** Flow-cytometric analysis of pro-B and pre-B cells in the bone marrow of 3–4-wk-old *Pax5*^+/+^, *Pax5*^∆OP/∆OP^, *Pax5*^∆HD/∆HD^, and *Pax5*^∆OP,HD/∆OP,HD^ mice. The percentage of cells in the indicated gates is shown. The gating strategy for defining the pro-B cells (B220^+^CD19^+^Kit^+^CD2^−^IgM^−^IgD^−^) and pre-B cells (B220^+^CD19^+^Kit^−^CD2^+^IgM^−^IgD^−^) is shown in Fig. S1 C. One of four to seven independent experiments is shown. **(C and D)** B cell numbers in the bone marrow (C) and spleen (D) of mice of the indicated genotypes at the ages of 3–4 (C) and 8–10 (D) wk, based on the flow-cytometric data shown in Fig. S1, C and D. Absolute numbers of total B, pro-B, pre-B, immature (imm) B, FO B, and MZ B cells are shown as mean values of four to seven independent experiments with SEM (*n* ≥ 8). Statistical data (C and D) were analyzed by one-way ANOVA with Dunnett’s multiple comparison test; *P < 0.05, **P < 0.01, ***P < 0.001, and ****P < 0.0001. Each dot (C and D) corresponds to one mouse. The definition of the different cell types is described in Fig. S1, C and D, and Materials and methods. **(E)** Scatter plot of gene expression differences between ex vivo sorted *Pax5*^+/+^ pro-B cells and *Pax5*^∆OP/∆OP^, *Pax5*^∆HD/∆HD^, or *Pax5*^∆OP,HD/∆OP,HD^ pro-B cells, respectively. The expression data of individual genes (dots) are plotted as mean normalized rlog (regularized logarithm) values and are based on two RNA-seq experiments per genotype (Table S1). Genes with an expression difference of greater than threefold, an adjusted P value of <0.05, and a mean TPM value of >5 in one pro-B cell type are colored in green or red, corresponding to activated or repressed genes, respectively. **(F)** Heatmap of gene expression differences in *Pax5*^∆OP/∆OP^, *Pax5*^∆HD/∆HD^, and *Pax5*^∆OP,HD/∆OP,HD^ pro-B cells relative to *Pax5*^+/+^ pro-B cells. The significantly differentially expressed genes are highlighted by a black box. The ratio of mRNA expression difference was determined for each gene by dividing the mean normalized rlog value of *Pax5*^+/+^ pro-B cells by the corresponding value of the *Pax5*^∆OP/∆OP^, *Pax5*^∆HD/∆HD^, or *Pax5*^∆OP,HD/∆OP,HD^ pro-B cells. Genes with decreased expression in the mutant pro-B cells compared with *Pax5*^+/+^ pro-B cells are marked in green (indicating activation by the OP or HD), while genes with increased expression in the mutant pro-B cells compared with *Pax5*^+/+^ pro-B cells are marked in red (indicating repression by the OP or HD). FC, fold change. **(G)** Expression of selected OP- and HD-dependent genes in *Pax5*^+/+^, *Pax5*^∆OP/∆OP^, *Pax5*^∆HD/∆HD^, and *Pax5*^∆OP,HD/∆OP,HD^ pro-B cells, shown as mean TPM values of two RNA-seq experiments per genotype. The RNA-binding protein hnRNPLL (encoded by *Hnrnpll*) regulates alternative splicing of the *Ptprc* (CD45) transcript (Oberdoerffer et al., 2008). At low hnRNPLL expression, the B cell–specific CD45 isoform B220 is generated, while higher hnRNPLL expression results in the expression of other CD45 isoforms at the expense of B220 (Jurado et al., 2022; Oberdoerffer et al., 2008). The derepression of *Hnrnpll* in *Pax5*^∆OP/∆OP^ and *Pax5*^∆OP,HD/∆OP,HD^ pro-B cells thus explains the specific downregulation of B220 expression on B cells of *Pax5*^∆OP/∆OP^ and *Pax5*^∆OP,HD/∆OP,HD^ mice (Fig. S1, C and D).

**Figure S1.**
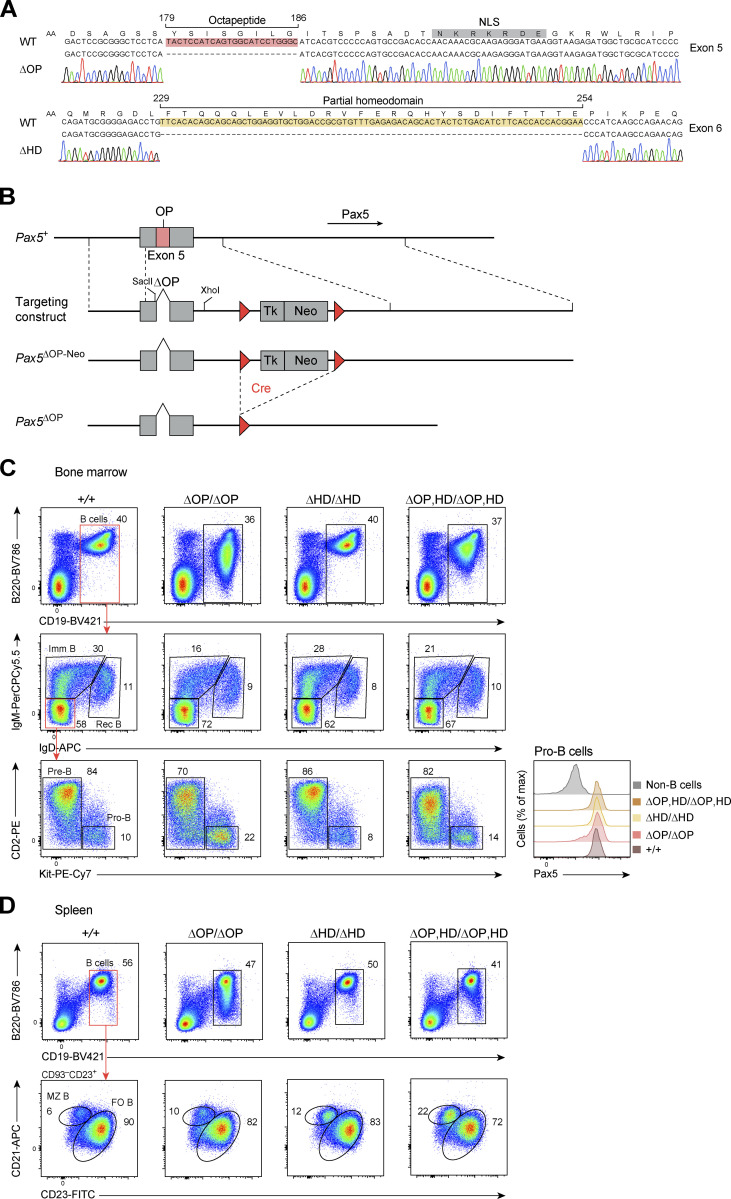
**Generation and B cell phenotype of the OP and HD deletions. (A)** Generation of the *Pax5*^∆OP^ and *Pax5*^∆HD^ alleles by deletion of the indicated DNA sequences in exon 5 or 6 of the endogenous mouse *Pax5* locus, respectively. The introduced deletion was verified by PCR amplification, cloning, and Sanger sequencing of genomic DNA from *Pax5*^∆OP/+^ and *Pax5*^∆HD/+^ mice, as shown by the respective sequencing chromatograms. The OP deletion was introduced by ES cell targeting, while the HD deletion was generated by CRISPR/Cas9-mediated mutagenesis in injected mouse zygotes (Yang et al., 2013; Materials and methods). **(B)** Generation of the *Pax5*^∆OP^ allele by ES cell targeting. The OP deletion was introduced by PCR amplification, and the corresponding PCR fragment was cloned between SacII and XhoI sites into the targeting vector containing 1.8- and 4.5-kb-long homology regions (indicated by dashed brackets). The neomycin (Neo) resistance gene was transcribed from the HSV thymidine kinase (Tk) promoter. Following blastocyst injection of correctly targeted ES cells and subsequent germline transmission, the *loxP*-flanked neomycin resistance cassette was deleted by Cre recombinase in the germline of *Pax5*^∆OP-Neo/+^ *Meox2*^Cre/+^ mice. *LoxP* sites are indicated by red arrowheads. **(C)** Flow-cytometric analysis of the indicated B cell types in the bone marrow of 3–4-wk-old *Pax5*^+/+^, *Pax5*^∆OP/∆OP^, *Pax5*^∆HD/∆HD^, and *Pax5*^∆OP,HD/∆OP,HD^ mice. Numbers refer to the percentage of cells in the indicated gate. Pax5 protein expression in pro-B cells (B220^+^CD19^+^Kit^+^CD2^−^IgM^−^IgD^−^) of the indicated genotypes was determined by flow-cytometric analysis of intracellular Pax5 staining (right). Immature, imm; Rec, recirculating. **(D)** Flow-cytometric analysis of FO and MZ B cells in the spleen of 8–10-wk-old mice of the indicated genotypes. The definition of the different cell types is described in Materials and methods. One of four to seven independent experiments (C and D) is shown.

To study the function of the conserved OP and HD of Pax5, we deleted these two sequence motifs individually or in combination in the endogenous *Pax5* locus by embryonic stem (ES) cell targeting (∆OP) or CRISPR/Cas9-mediated mutagenesis (∆HD and ∆OP,HD) in mouse zygotes (Yang et al., 2013; Fig. 2 A; and Fig. S1, A and B). Flow-cytometric analyses revealed that pre-B, immature B, and total B cells were moderately reduced in the bone marrow of *Pax5*^∆OP/∆OP^ mice compared with control *Pax5*^+/+^ mice (Fig. 2, B and C; and Fig. S1 C). In contrast, the *Pax5*^∆OP/∆OP^ pro-B cells were 1.7-fold increased relative to *Pax5*^+/+^ pro-B cells (Fig. 2, B and C), indicating that loss of the OP resulted in a partial arrest of B cell development at the pro-B to pre-B cell transition (Fig. 2, B and C). The pro-B cells of *Pax5*^∆HD/∆HD^ mice were, however, 2.1-fold decreased compared with *Pax5*^+/+^ pro-B cells, leading to reduced numbers of pre-B, immature B, and total B cells in the bone marrow of *Pax5*^∆HD/∆HD^ mice compared with *Pax5*^+/+^ mice (Fig. 2, B and C; and Fig. S1 C). Interestingly, simultaneous deletion of the OP and HD revealed an additive effect of these mutations on the generation of each B cell type in the bone marrow (Fig. 2 C). This effect is best shown for pro-B cells as the increase observed for *Pax5*^∆OP/∆OP^ pro-B cells was equalized by the decrease seen for *Pax5*^∆HD/∆HD^ pro-B cells, thus resulting in similar numbers of pro-B cells in *Pax5*^∆OP,HD/∆OP,HD^ as in *Pax5*^+/+^ mice (Fig. 2 C and Fig. S1 C). An additive effect was also observed for splenic B cells as there was a gradual reduction of total and FO B cells from *Pax5*^∆OP/∆OP^ mice to *Pax5*^∆HD/∆HD^ mice, resulting in a 2.4-fold loss of these B cells in *Pax5*^∆OP,HD/∆OP,HD^ mice (Fig. 2 D and Fig. S1 D). In contrast, a gradual increase of marginal zone (MZ) B cells was observed for both mutations, leading to a 1.7-fold increase of MZ B cells in the spleen of *Pax5*^∆OP,HD/∆OP,HD^ mice relative to *Pax5*^+/+^ mice (Fig. 2 D and Fig. S1 D). Deletion of the two central domains did not, however, affect expression of the Pax5 protein, as shown by flow-cytometric analysis of intracellular Pax5 staining in pro-B cells (Fig. S1 C). Hence, these data indicate that the OP and HD of Pax5 are essential for optimal B cell development.

### Distinct roles of the Pax5 OP and HD in gene repression and activation

**Figure S2.**
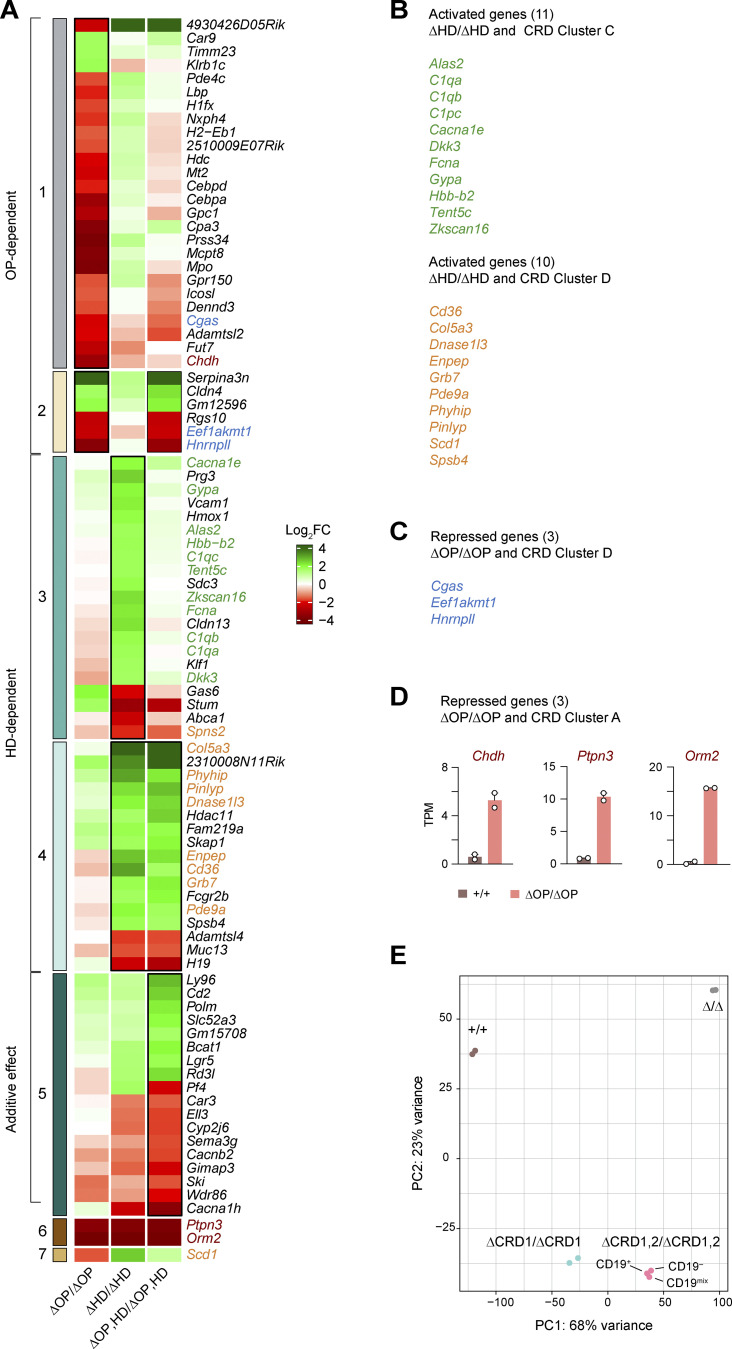
**Heatmap of differential gene expression in *Pax5***^**∆OP/∆OP**^**, *Pax5***^**∆HD/∆HD**^**, and *Pax5***^**∆OP,HD/∆OP,HD**^ **pro-B cells at higher magnification. (A)** The heatmap of the differentially expressed genes, shown in Fig. 2 F, is enlarged to show the expression of the individual genes in *Pax5*^∆OP/∆OP^, *Pax5*^∆HD/∆HD^, and *Pax5*^∆OP,HD/∆OP,HD^ pro-B cells relative to *Pax5*^+/+^ pro-B cells. For further information, see the legend of Fig. 2 F. **(B)** Activated genes that were similarly deregulated in *Pax5*^∆HD/∆HD^ pro-B cells as well as in *Pax5*^∆CRD1/−^ and *Pax5*^∆CRD1,2/−^ pro-B cells (with the indicated genes belonging to the CRD-regulated gene cluster C (green) or D (orange; Table S2). **(C)** The genes *Cgas*, *Eef1akmt1*, and *Hnrnpll* (blue) were equally depressed in *Pax5*^∆OP/∆OP^ pro-B cells and *Pax5*^∆CRD1/–^, *Pax5*^∆CRD2/–^, and *Pax5*^∆CRD1,2/–^ pro-B cells (belonging to the CDR-regulated gene cluster D; Fig. 5 D and Fig. S4 D). **(D)** The genes *Chdh*, *Ptpn3*, and *Orm2* are derepressed in *Pax5*^∆OP/∆OP^ pro-B cells (red), but are not expressed in *Pax5*^∆CRD1/−^, *Pax5*^∆CRD2/−^, and *Pax5*^∆CRD1,2/−^ pro-B cells as they belonging to the CRD-regulated gene cluster A (Fig. 5 D and Fig. S4 D). The expression of *Chdh*, *Ptpn3*, and *Orm2* in *Pax5*^+/+^ and *Pax5*^∆OP/∆OP^ pro-B cells is shown as mean TPM values of two RNA-seq experiments per genotype. **(E)** Principal component analysis based on open chromatin data that were generated by ATAC-seq analysis of ex vivo sorted *Pax5*^+/+^, *Pax5*^∆CRD1/∆CRD1^, *Pax5*^∆CRD1,2/∆CRD1,2^ pro-B, and *Pax5*^∆/∆^ (*Vav*-Cre *Pax5*^fl/fl^) progenitor cells of the bone marrow. CD19^–^, CD19^+^, and unfractionated CD19^mix^ cells were sorted as Lin^–^Ly6D^+^B220^+^Kit^hi^ cells from the bone marrow prior to ATAC-seq analysis.

We next investigated the effect of loss of the central Pax5 domains on gene expression by performing RNA sequencing (RNA-seq) with ex vivo sorted *Pax5*^+/+^, *Pax5*^∆OP/∆OP^, *Pax5*^∆HD/∆HD^, and *Pax5*^∆OP,HD/∆OP,HD^ pro-B cells. For the analysis of pairwise comparisons, we defined differentially expressed genes as significantly increased or decreased in the *Pax5* mutant pro-B cells relative to *Pax5*^+/+^ pro-B cells, if they exhibited an expression difference of greater than threefold, an adjusted P value of <0.05 and a mean expression value of >5 transcripts per million (TPM) in one pro-B cell type (Table S1). The gene expression differences are displayed as a scatter plot in Fig. 2 E, while the overlap between the differentially expressed genes is shown as a heatmap, resulting in seven distinct clusters (Fig. 2 F and Fig. S2 A). A similar number of differentially expressed genes were identified by comparing *Pax5*^+/+^ and *Pax5*^∆OP/∆OP^ pro-B cells (35), *Pax5*^+/+^, and *Pax5*^∆HD/∆HD^ pro-B cells (41) as well as *Pax5*^+/+^ and *Pax5*^∆OP,HD/∆OP,HD^ pro-B cells (43; Fig. 2 E and Fig. S2 A; and Table S1). Notably, the majority (83%) of the 35 deregulated genes in *Pax5*^∆OP/∆OP^ pro-B cells were upregulated, indicating that the OP primarily mediates gene repression (Fig. 2, E–G; and Fig. S2 A). In contrast, the expression of most (78%) of the 41 deregulated genes was decreased in *Pax5*^∆HD/∆HD^ pro-B cells, demonstrating that the HD predominantly contributes to gene activation (Fig. 2, E–G; and Fig. S2, A–D). Hence, the OP and HD of Pax5 have different functions in transcriptional regulation, which was further corroborated by the fact that only two genes (*Orm2* and *Ptpn3*; cluster 6) were similarly regulated in both *Pax5*^∆OP/∆OP^ and *Pax5*^∆HD/∆HD^ pro-B cells (Fig. S2 A). Consistent with independent functions of the two central domains, the numbers of activated (58%) and repressed (42%) genes were more balanced in the double-mutant *Pax5*^∆OP,HD/∆OP,HD^ pro-B cells (Fig. 2 E and Fig. S2 A). Notably, 6 (cluster 2) of the 32 OP-dependent genes in *Pax5*^∆OP/∆OP^ pro-B cells and 17 (cluster 4) of the 38 HD-dependent genes in *Pax5*^∆HD/∆HD^ pro-B cells were similarly regulated in the double-mutant *Pax5*^∆OP,HD/∆OP,HD^ pro-B cells (Fig. 2, F and G; and Fig. S2 A). Moreover, 18 genes (cluster 5) reached a statistically significant difference of gene expression only in *Pax5*^∆OP,HD/∆OP,HD^ pro-B cells as a result of additive regulatory effects of the OP and HD (Fig. 2, F and G; and Fig. S2 A). In summary, our molecular data identified a critical role of the OP and HD of Pax5 in gene repression and activation, respectively.

### Essential role of the C-terminal Pax5 domains in controlling B cell development

**Figure 3.**
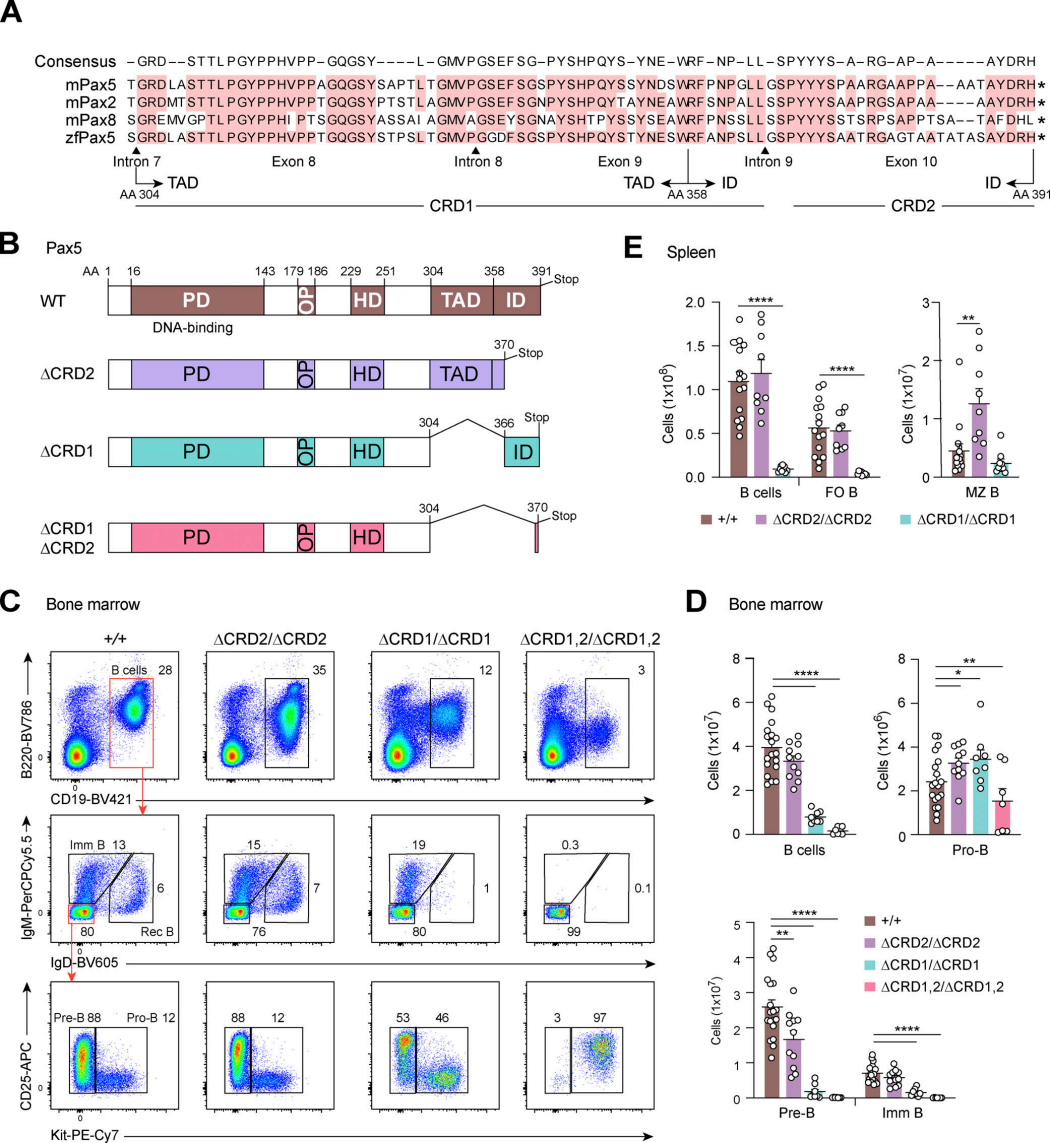
**Essential role of the C-terminal Pax5 domains in controlling B cell development. (A)** Strong conservation of the C-terminal sequences of the mouse (m) Pax2, Pax5, Pax8, and zebrafish (zf) Pax5 proteins (Dörfler and Busslinger, 1996; Pfeffer et al., 1998). The extent of the previously described TAD and ID (Dörfler and Busslinger, 1996) are indicated together with the respective exons, introns, and amino acid positions of Pax5. CRD1 is defined by the amino acid sequence encoded by *Pax5* exons 8 and 9, while CRD2 consists of the indicated amino acid sequence encoded by exon 10. The stop codon is denoted by an asterisk. **(B)** Domain organization of Pax5 indicating the extent of sequence deletion present in the *Pax5*^∆CRD1^, *Pax5*^∆CRD2^, and *Pax5*^∆CRD1,2^ alleles, as shown in A. PD, paired domain. **(C)** Flow-cytometric analysis of the indicated B cell types in the bone marrow of 3–5-wk-old *Pax5*^+/+^, *Pax5*^∆CRD2/∆CRD2^, *Pax5*^∆CRD1/∆CRD1^, and *Pax5*^∆CRD1,2/∆CRD1,2^ mice. The percentage of cells in the indicated gates is shown. The downregulation of B220 on the *Pax5* mutant cells is caused by derepression of the Pax5-repressed gene *Hnrnpll* (Fig. 5 G), as explained in the legend of Fig. 2 G. One of six independent experiments is shown. Imm, immature; Rec, recirculating. **(D and E)** B cell numbers in the bone marrow (D) and spleen (E) of mice of the indicated genotypes, determined by the flow-cytometric data shown in C or Fig. S3 C (spleen). Absolute numbers of total B, pro-B, pre-B, imm B, FO B, and MZ B cells are shown as mean values of six independent experiments with SEM (*n* ≥ 7). Statistical data (D and E) were analyzed by one-way ANOVA with Dunnett’s multiple comparison test; *P < 0.05, **P < 0.01, and ****P < 0.0001. Each dot (D and E) corresponds to one mouse.

**Figure S3.**
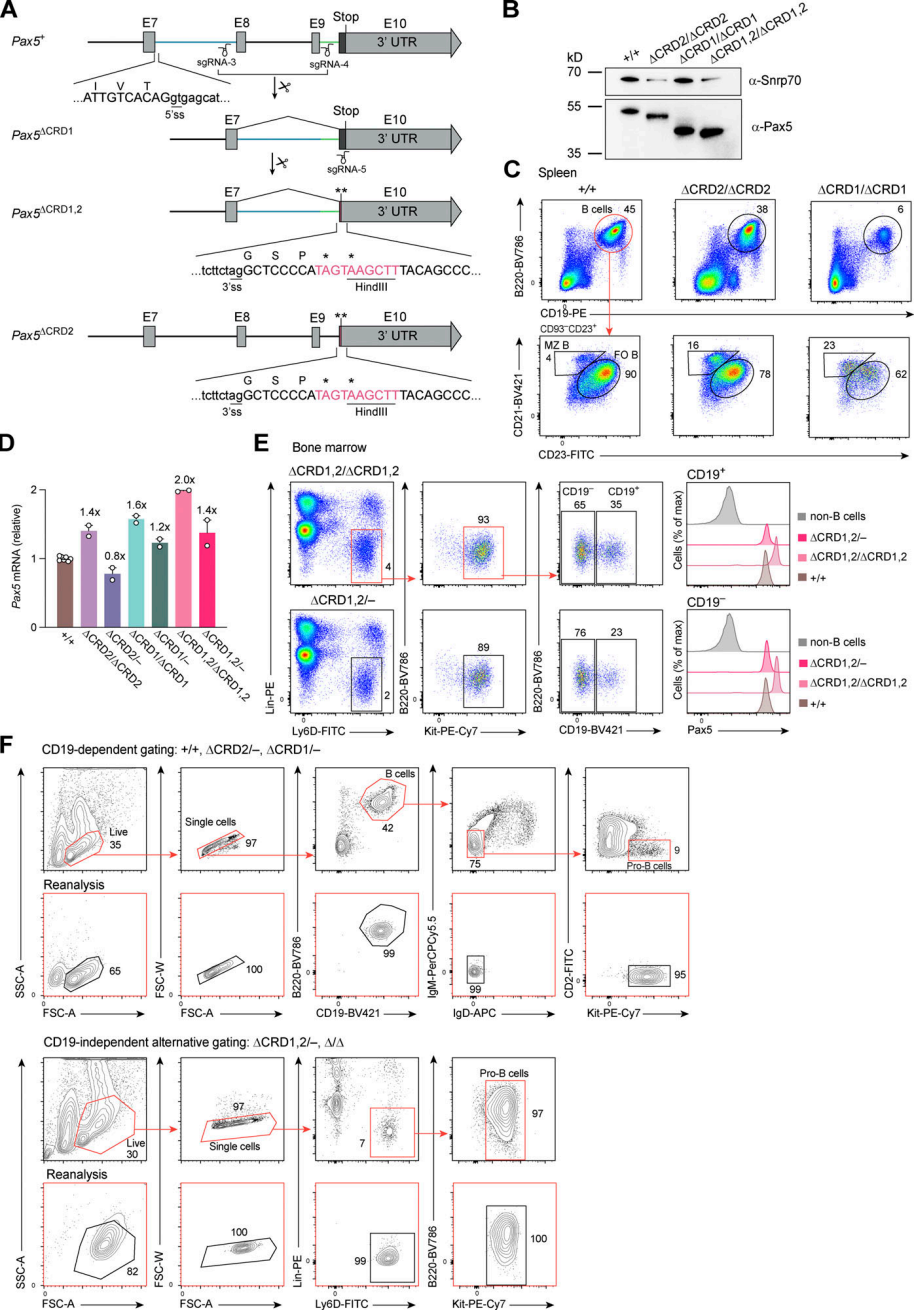
**Generation and characterization of the *Pax5***^**∆CRD1**^**, *Pax5***^**∆CRD2**^**, and *Pax5***^**∆CRD1,2**^ **alleles. (A)** Generation of the *Pax5*^∆CRD1^, *Pax5*^∆CRD2^, and *Pax5*^∆CRD1,2^ alleles. The *Pax5*^∆CRD1^ allele was created by deletion of genomic sequences from *Pax5* exon 8 to exon 9 by CRISPR/Cas9-mediated DNA cleavage with sgRNA-3 and sgRNA-4 targeting intronic sequences located upstream of exon 8 and downstream of exon 9, respectively (Table S6 and Materials and methods). The coding sequences at the 5′ end of exon 10 (Fig. 3 A) were deleted in the *Pax5*^+^ or *Pax5*^∆CRD1^ allele by using sgRNA-5 and a corresponding repair template (Table S6) to generate the *Pax5*^∆CRD2^ and *Pax5*^∆CRD1,2^ alleles, respectively. The exon–intron junctions containing the 5′ splice site (5′ss) of exon 7 and 3′ splice site (3′ss) of exon 10 are indicated together with the newly inserted DNA sequence (red) containing two stop codons (*) and a HindIII site in exon 10. UTR, untranslated region. **(B)** Immunoblot analysis of whole-cell extracts from in vitro cultured *Pax5*^∆CRD2/∆CRD2^, *Pax5*^∆CRD1/∆CRD1^, and *Pax5*^∆CRD1,2/∆CRD1,2^ pro-B cells with anti-Pax5 and anti-Snrp70 antibodies. The Snrp70 protein was analyzed as loading control, and marker proteins of the indicated size (in kilodaltons, kD) are shown. One of two independent experiments is shown. **(C)** Flow-cytometric analysis of FO and MZ B cells in the spleen of *Pax5*^+/+^, *Pax5*^∆CRD2/∆CRD2^, and *Pax5*^∆CRD1/∆CRD1^ mice at the age of 8–10 wk. Numbers refer to the percentage of cells in the indicated gate. One of six independent experiments is shown. **(D)** RNA-seq analysis of *Pax5* mRNA expression, which is shown as the mean TPM value for pro-B cells of the indicated genotypes relative to that of control *Pax5*^+/+^ pro-B cells (set to 1). **(E)** Flow-cytometric analysis of B cell progenitors in the bone marrow of *Pax5*^∆CRD1,2/∆CRD1,2^ and *Pax5*^∆CRD1,2/−^ mice. Intracellular staining revealed Pax5 protein expression in both CD19^−^ and CD19^+^ populations of the indicated B cell progenitors (Lin^−^Ly6D^+^B220^+^Kit^hi^). One of three independent experiments is shown. **(F)** Flow-cytometric sorting of *Pax5*^∆CDR1/−^ pro-B cells (upper part) and *Pax5*^∆CRD1,2/−^ progenitors (lower part) from the bone marrow of mice at the age of 3–5 wk. The different gates used for flow-cytometric sorting are indicated. The purity of the sorted cell populations was determined by flow cytometric reanalysis (lower row). The percentage of cells in the different gates is shown. The *Pax5*^+/+^ and *Pax5*^∆CDR2/−^ pro-B cells were sorted as shown for the *Pax5*^∆CDR1/−^ pro-B cells (upper part), while the *Pax5*^∆/∆^ (*Vav*-Cre *Pax5*^fl/fl^) progenitors were sorted like the *Pax5*^∆CRD1,2/−^ progenitors (lower part). Source data are available for this figure: SourceData FS3.

The C-terminal sequences of Pax5, which are highly conserved in the mouse Pax2, Pax8, and zebrafish Pax5 proteins, consist of the TAD and ID that we previously identified by transient transfection experiments in established B cell lines (Dörfler and Busslinger, 1996; Fig. 3 A). For our in vivo study in the mouse, we divided the C-terminal sequences into a C-terminal regulatory domain 1 (CRD1), corresponding to *Pax5* exons 8 and 9 encoding the TAD with a small C-terminal extension, and a C-terminal regulatory domain 2 (CRD2), consisting of an N-terminally shortened version of the ID encoded by exon 10 (Fig. 3, A and B). We used CRISPR/Cas9-mediated mutagenesis in mouse zygotes to generate the *Pax5*^∆CRD1^, *Pax5*^∆CRD2^, and double-mutant *Pax5*^∆CRD1,2^ alleles (Fig. 3 B and Fig. S3 A). Immunoblot analysis of *Pax5*^∆CRD2/∆CRD2^, *Pax5*^∆CRD1/∆CRD1^, *Pax5*^∆CRD1,2/∆CRD1,2^, and *Pax5*^+/+^ pro-B cells revealed that mutant Pax5 proteins of the correct size were expressed in the corresponding pro-B cells (Fig. S3 B), indicating that the mRNA splicing to the respective exons (Fig. S3 A) was not affected by the genetic manipulations. Flow-cytometric analyses of the bone marrow and spleen of *Pax5*^∆CRD2/∆CRD2^, *Pax5*^∆CRD1/∆CRD1^, and *Pax5*^∆CRD1,2/∆CRD1,2^ mice revealed the following B cell developmental phenotypes (Fig. 3, C–E; and Fig. S3 C). First, deletion of CRD2 minimally affected B cell development in *Pax5*^∆CRD2/∆CRD2^ mice compared with *Pax5*^+/+^ mice (Fig. 3, C–E). The pro-B cells were 1.5-fold increased, while the pre-B and immature B cells were moderately decreased in the bone marrow of *Pax5*^∆CRD2/∆CRD2^ mice relative to *Pax5*^+/+^ mice, thus indicating a minor block of B cell development at the pro-B to pre-B cell transition (Fig. 3 D). Notably, deletion of CRD2 resulted in a 2.8-fold increase of splenic MZ B cells but did not affect the generation of FO B cells in *Pax5*^∆CRD2/∆CRD2^ mice (Fig. 3 E and Fig. S3 C). Second, elimination of CRD1 strongly interfered with B lymphopoiesis, as only pro-B cells were 1.6-fold increased, whereas all other B cell types were decreased in number in the bone marrow and spleen of *Pax5*^∆CRD1/∆CRD1^ mice compared with *Pax5*^+/+^ mice (Fig. 3, C–E; and Fig. S3 C). Third, elimination of both CRD1 and CRD2 was only compatible with the generation of reduced numbers of pro-B cells as pre-B cells and all subsequent B cell developmental stages were absent in *Pax5*^∆CRD1,2/∆CRD1,2^ mice (Fig. 3, C and D). We conclude therefore that normal B cell development strictly depends on the C-terminal regulatory sequences of Pax5.

### Increased expression of the Pax5 protein upon deletion of the C-terminal domains

**Figure 4.**
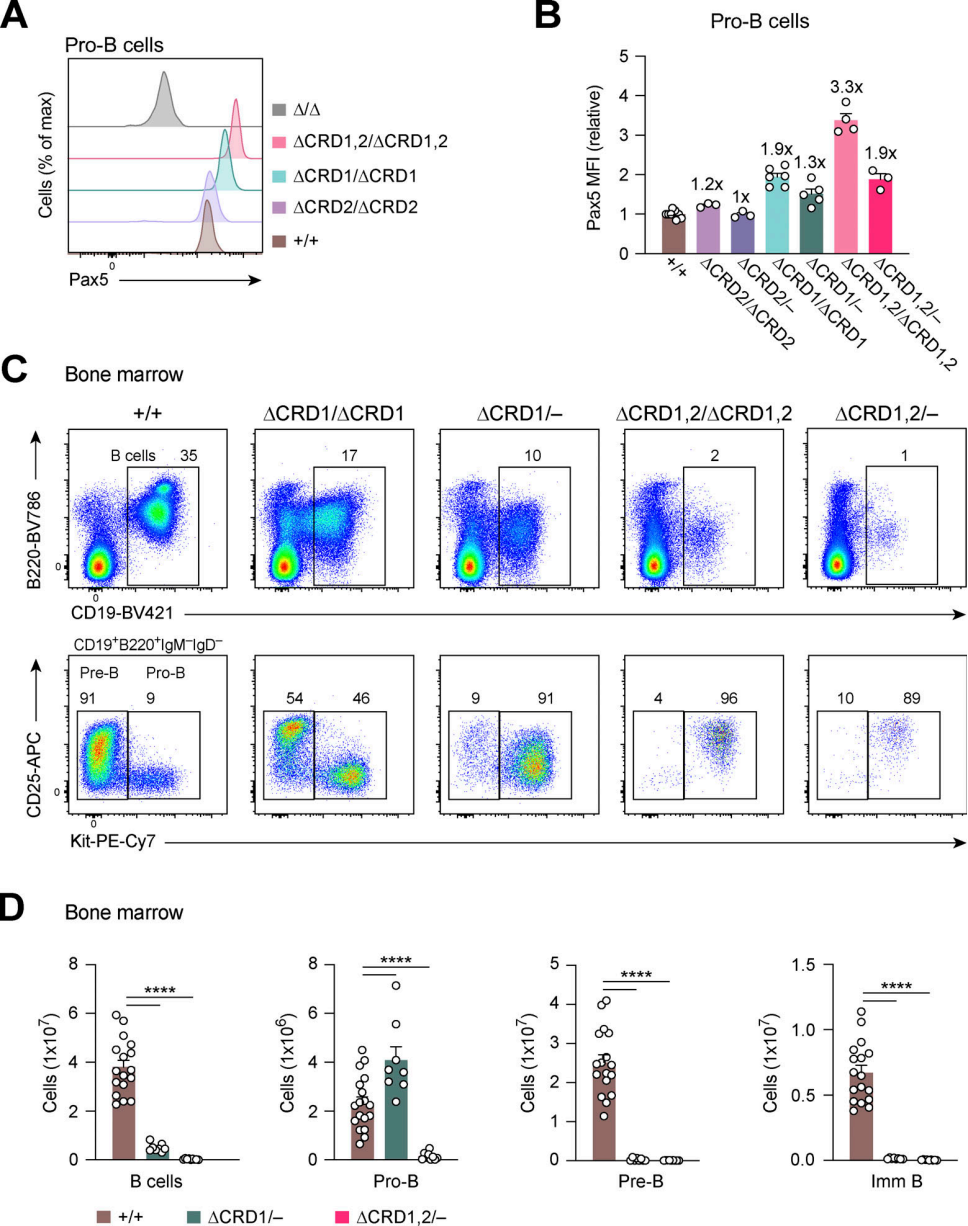
**Dosage-sensitive B cell phenotype upon deletion of C-terminal Pax5 domains. (A)** Pax5 expression in pro-B cells from the bone marrow of *Pax5*^+/+^, *Pax5*^∆CRD2/∆CRD2^, *Pax5*^∆CRD1/∆CRD1^, *Pax5*^∆CRD1,2/∆CRD1,2^, and *Pax5*^∆/∆^ (*Vav*-Cre *Pax5*^fl/fl^) mice. Pax5 protein levels were determined by flow-cytometric analysis of intracellular Pax5 staining. One of five independent experiments is shown. **(B)** Relative measurement of Pax5 protein expression in pro-B cells of the indicated genotypes by intracellular Pax5 staining. The geometric mean fluorescence intensity (MFI) was determined for pro-B cells of the indicated genotypes relative to that of control *Pax5*^+/+^ pro-B cells (set to 1) and is shown a mean value of five independent experiments with SEM. **(C)** Flow-cytometric analysis of total B, pro-B, and pre-B cells in the bone marrow of 3–5-wk-old *Pax5*^+/+^, *Pax5*^∆CRD1/∆CRD1^, *Pax5*^∆CRD1/−^, *Pax5*^∆CRD1,2/∆CRD1,2^, and *Pax5*^∆CRD1,2/−^ mice. The percentage of cells in the indicated gates is shown. Note that *Il2ra* (CD25) is a repressed Pax5 target gene (Revilla-i-Domingo et al., 2012) that is no longer repressed in the CD25^+^ pro-B cells of *Pax5*^∆CRD1,2/∆CRD1,2^ and *Pax5*^∆CRD1,2/−^ mice. The downregulation of B220 on the *Pax5* mutant cells is caused by derepression of the Pax5-repressed gene *Hnrnpll* (Fig. 5 G), as explained in the legend of Fig. 2 G. One of five independent experiments is shown. **(D)** B cell numbers in the bone marrow of mice of the indicated genotypes, determined by the flow-cytometric data shown in C. Absolute numbers of total B, pro-B, pre-B, and immature (imm) B cells are shown as mean values of five independent experiments with SEM (*n* ≥ 8). Statistical data were analyzed by one-way ANOVA with Dunnett’s multiple comparison test; ****P < 0.0001. Each dot (D) corresponds to one mouse.

We next analyzed the expression of the C-terminally mutated Pax5 proteins in pro-B cells from *Pax5*^∆CRD2/∆CRD2^, *Pax5*^∆CRD1/∆CRD1^, and *Pax5*^∆CRD1,2/∆CRD1,2^ mice by intracellular Pax5 staining. Interestingly, Pax5 protein expression was gradually upregulated with increasing deletion of the C-terminal sequences from Pax5-∆CRD2 (1.2-fold) to Pax5-∆CRD1 (1.9-fold) and Pax5-∆CRD1,2 (3.3-fold) relative to the full-length Pax5 protein (Fig. 4, A and B). *Pax5* mRNA expression was also increased in pro-B cells of *Pax5*^∆CRD2/∆CRD2^, *Pax5*^∆CRD1/∆CRD1^, and *Pax5*^∆CRD1,2/∆CRD1,2^ mice (Fig. S3 D), suggesting that the C-terminal domains of Pax5 may be involved in moderate auto-repression of the *Pax5* gene. Based on these data, it is conceivable that, in addition to the absence of the C-terminal regulatory sequences, the increased expression of the mutant Pax5 proteins may also contribute to the observed phenotype of the *Pax5*^∆CRD2/∆CRD2^, *Pax5*^∆CRD1/∆CRD1^, and *Pax5*^∆CRD1,2/∆CRD1,2^ mice. To test this hypothesis, we replaced one mutant *Pax5* allele with the *Pax5* null allele (Urbánek et al., 1994) to lower the expression of the C-terminally truncated Pax5 proteins. As shown by intracellular staining, the Pax5 expression levels were reduced and thus more normalized in *Pax5*^∆CRD2/−^, *Pax5*^∆CRD1/−^, and *Pax5*^∆CRD1,2/−^ pro-B cells (Fig. 4 B). This in turn increased the severity of the respective B cell phenotype. The few pre-B and immature B cells that were still generated in the bone marrow of *Pax5*^∆CRD1/∆CRD1^ mice (Fig. 3, C and D) were lost in *Pax5*^∆CRD1/−^ mice (Fig. 4, C and D). Moreover, the pro-B cells were further decreased in *Pax5*^∆CRD1,2/−^ mice (Fig. 4, C and D) compared with *Pax5*^∆CRD1,2/∆CRD1,2^ mice (Fig. 3, C and D). Interestingly, the pro-B cells of *Pax5*^∆CRD1,2/∆CRD1,2^ and *Pax5*^∆CRD1,2/−^ mice strongly expressed CD25 (Fig. 4 C), which is encoded by the repressed Pax5 target gene *Il2ra* (Revilla-i-Domingo et al., 2012; Table S2), thus suggesting that the pro-B cells of these two genotypes may resemble Pax5-deficient progenitors in this regard. By defining the pro-B cells of *Pax5*^∆CRD1,2/∆CRD1,2^ and *Pax5*^∆CRD1,2/−^ mice in a CD19-independent manner as Lin^−^Ly6D^+^B220^+^Kit^hi^ cells, we realized that these cells were heterogeneous as they consisted of a larger CD19^−^Pax5^+^ and smaller CD19^+^Pax5^+^ cell population (Fig. S3 E). However, the CD19^−^ and CD19^+^ cell fractions of the *Pax5*^∆CRD1,2/∆CRD1,2^ pro-B cells clustered closely together and were clearly separated from *Pax5*^∆/∆^ (*Vav*-Cre *Pax5*^fl/fl^) progenitors by principal component analysis (Fig. S2 E), which was based on open chromatin data generated by the assay for transposase-accessible chromatin (ATAC) coupled with deep sequencing (ATAC-seq; Buenrostro et al., 2013; see below). We conclude therefore that *Pax5*^∆CRD1,2/∆CRD1,2^ pro-B cells are arrested at a different developmental stage compared with *Pax5*^∆/∆^ progenitors.

### Pax5 regulates gene activation and repression largely through its C-terminal domains

**Figure 5.**
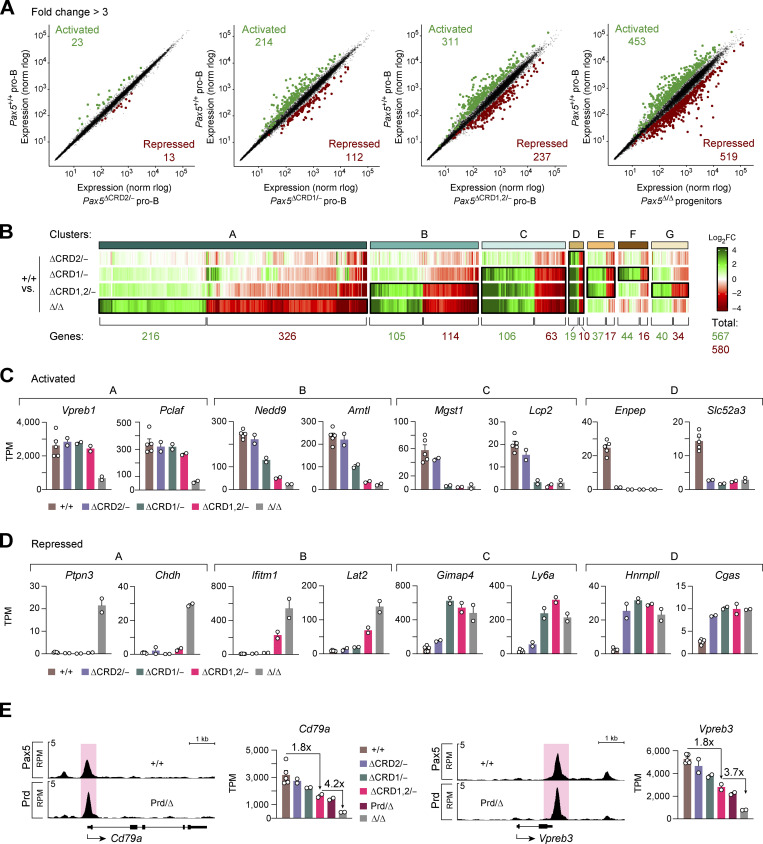
**Role of the C-terminal Pax5 domains in gene activation and repression. (A)** Scatter plot of gene expression differences between ex vivo sorted *Pax5*^+/+^ pro-B cells and *Pax5*^∆CRD2/−^, *Pax5*^∆CRD1/−^, *Pax5*^∆CRD1,2/−^ pro-B cells, or *Pax5*^∆/∆^ (*Vav*-Cre *Pax5*^fl/fl^) progenitor cells, respectively. The expression data of individual genes (dots) are plotted as mean normalized rlog (regularized logarithm) values and are based on two RNA-seq experiments per genotype except for the five RNA-seq experiments performed with *Pax5*^+/+^ pro-B cells (Table S2). Genes with an expression difference of greater than threefold, an adjusted P value of <0.05, and a mean TPM value of >5 in one pro-B cell type are colored in green or red, corresponding to activated or repressed genes, respectively. The transgenic *Vav*-Cre line (de Boer et al., 2003) initiates Cre-mediated deletion of the floxed *Pax5* allele in the hematopoietic stem cells, thus leading to *Pax5* inactivation in the entire hematopoietic system (de Boer et al., 2003). **(B)** Heatmap of gene expression differences in *Pax5*^∆CRD2/−^, *Pax5*^∆CRD1/−^, *Pax5*^∆CRD1,2/−^ pro-B cells and *Pax5*^∆/∆^ progenitors relative to *Pax5*^+/+^ pro-B cells (see also Table S2). The significantly differentially expressed genes are highlighted by a black box. Genes with decreased expression in the mutant pro-B cells compared with *Pax5*^+/+^ pro-B cells are marked in green, while genes with increased expression in the mutant pro-B cells compared with *Pax5*^+/+^ pro-B cells are marked in red. FC, fold change. **(C and D)** Expression of selected activated (C) or repressed (D) genes belonging to the indicated gene expression clusters. The expressions of these genes in *Pax5*^+/+^, *Pax5*^∆CRD2/−^, *Pax5*^∆CRD1/−^, *Pax5*^∆CRD1,2/−^ pro-B cells, and *Pax5*^∆/∆^ progenitors are shown as mean TPM values of two or five RNA-seq experiments per genotype. The equal importance of CRD1 and CRD2 for the expression of cluster D genes likely points to a critical function of the amino acid sequences at the CRD1–CRD2 junction for the regulation of these genes. **(E)** Activation of the cluster A genes *Cd79a* and *Vpreb3* by the Pax5 Prd alone in *Pax5*^Prd/∆^ progenitor cells (right). The binding of Pax5 or the Prd polypeptide at the promoter of *Cd79a* and a downstream enhancer of *Vpreb3* is shown by ChIP-seq analysis of *Pax5*^+/+^ pro-B or *Pax5*^Prd/∆^ progenitor cells, respectively (left).

To gain insight into the gene-regulatory function of the C-terminal domains of Pax5, we performed RNA-seq experiments with ex vivo sorted *Pax5*^∆CRD1/−^, *Pax5*^∆CRD2/−^, and *Pax5*^∆CRD1,2/−^ pro-B cells as well as with control *Pax5*^+/+^ pro-B cells and *Pax5*^∆/∆^ progenitors (Fig. 5 A and Table S2). To this end, we isolated pro-B cells from *Pax5*^+/+^, *Pax5*^∆CRD1/−^, and *Pax5*^∆CRD2/−^ mice as B220^+^CD19^+^Kit^+^CD2^−^IgM^−^IgD^−^ cells by flow-cytometric sorting, while the CD19^low/−^ pro-B cells from *Pax5*^∆CRD1,2/−^ mice and CD19^−^ progenitors from *Pax5*^∆/∆^ mice were sorted as Lin^−^Ly6D^+^Kit^hi^B220^+^ cells (Fig. S3 F). For pairwise analysis of the RNA-seq data, we used the same cutoffs (greater than threefold deregulation) as described above for the analysis of *Pax5*^∆OP/∆OP^ and *Pax5*^∆HD/∆HD^ pro-B cells. Importantly, these gene expression analyses demonstrated that the CRD1 and CRD2 domains mediate both gene activation and repression (Fig. 5 A). As expected, these analyses also revealed that the phenotypic severity of the different *Pax5* mutant pro-B cells (Fig. 3, C–E; and Fig. 4 C) correlated with the number of differentially expressed genes, as only 36 genes were deregulated in the *Pax5*^∆CRD2/−^ pro-B with the weakest B cell phenotype compared with 548 genes in the *Pax5*^∆CRD1,2/−^ pro-B cells with the strongest phenotype (Fig. 5 A). An even greater number (972) of genes was deregulated in *Pax5*^∆/∆^ progenitors compared with *Pax5*^+/+^ pro-B cells (Fig. 5 A). This additional large increase of differentially expressed genes in *Pax5*^∆/∆^ progenitors could be caused by the arrest of these cells at an earlier developmental stage or by the complete absence of the Pax5 protein.

### Differential dependency of Pax5-regulated genes on the function of the C-terminal domains

**Figure S4.**
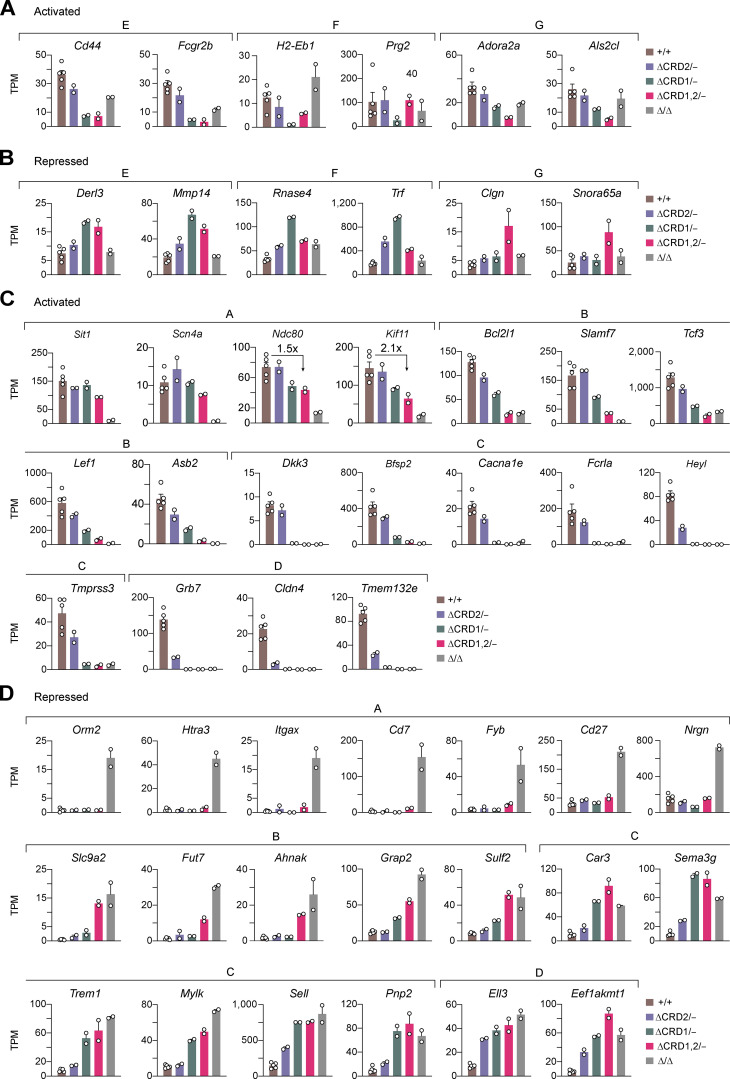
**Differential dependency of Pax5-regulated genes on the function of the C-terminal domains. (A–D)** Expression of selected genes in *Pax5*^+/+^, *Pax5*^∆CRD2/−^, *Pax5*^∆CRD1/−^, *Pax5*^∆CRD1,2/−^ pro-B cells, and *Pax5*^∆/∆^ progenitors is shown as mean TPM values of two or five RNA-seq experiments per genotype. **(A and B)** Pax5-activated (A) and Pax5-repressed (B) genes belonging to the gene expression clusters E, F, and G, as classified in Fig. 5 B. **(C and D)** Pax5-activated (C) and Pax5-repressed (D) genes belonging to the clusters A–D, as defined in Fig. 5 B.

We next compared the expression of the differentially regulated genes among the *Pax5*^∆CRD2/−^, *Pax5*^∆CRD1/−^, *Pax5*^∆CRD1,2/−^ pro-B cells and *Pax5*^∆/∆^ progenitors relative to *Pax5*^+/+^ pro-B cells. This analysis resulted in seven distinct gene clusters (A–G), as shown by the heatmap in Fig. 5 B (see also Table S2). Regulation of the genes present in the small clusters E, F, and G is unusual for the following reasons. While genes in these three clusters depended for their activation or repression on CRD1 (F) or both CRD1 and CRD2 (E and G), they exhibited the same common trait as they were expressed at similar levels in *Pax5*^∆/∆^ progenitors and *Pax5*^+/+^ pro-B cells (Fig. 5 B and Fig. S4, A and B). Moreover, genes of cluster F were deregulated predominantly upon deletion of CRD1 in *Pax5*^∆CRD1/−^ pro-B cells but not upon deletion of both CRD1 and CRD2 in *Pax5*^∆CRD1,2/−^ pro-B cells (Fig. 5 B and Fig. S4, A and B). As these two features are difficult to explain, we did not further analyze genes belonging to clusters E, F, and G.

**Figure 6.**
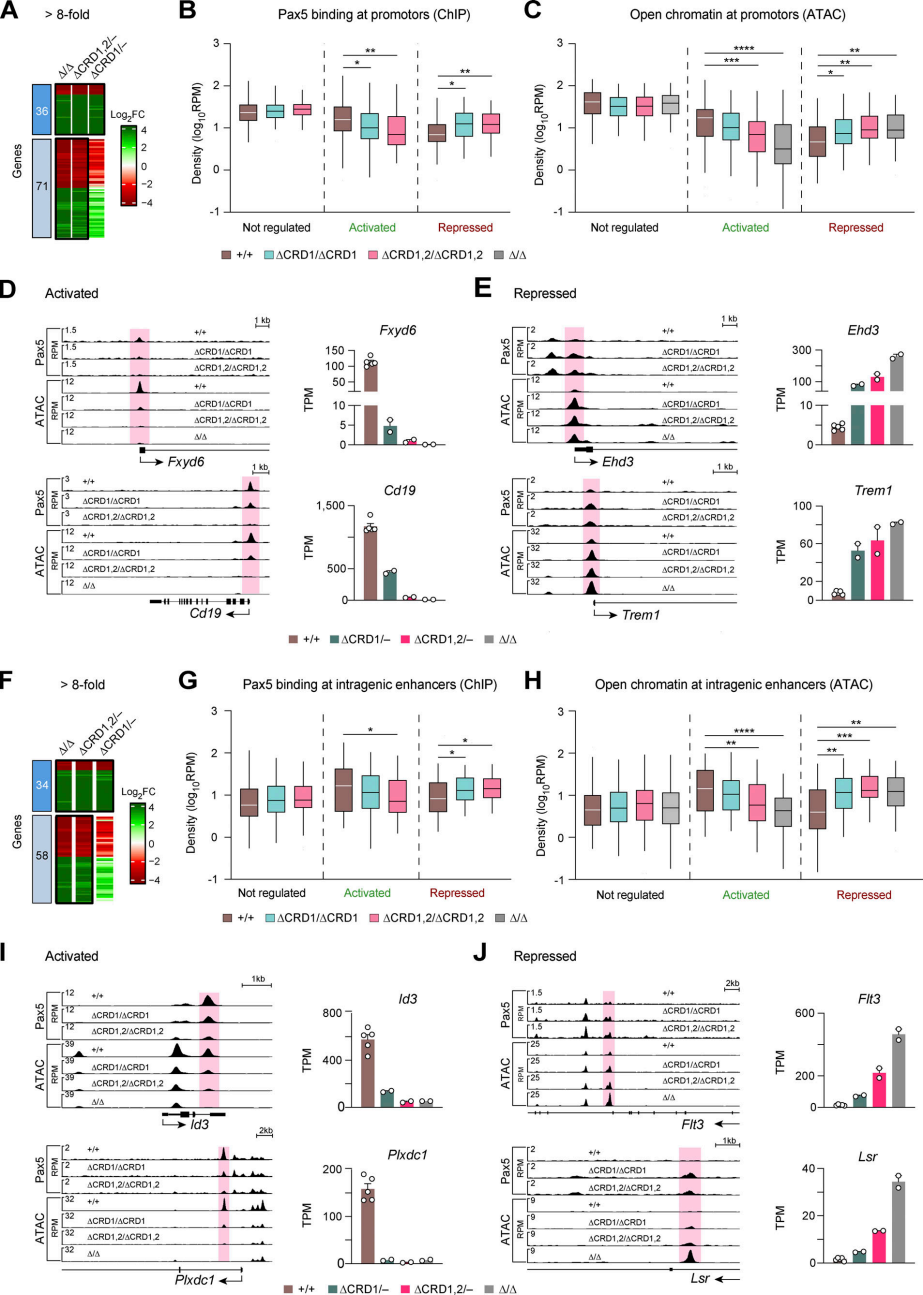
**Control of chromatin accessibility at promoters and intragenic enhancers of genes that are regulated by the C-terminal domains of Pax5. (A and F)** Heatmap of genes with a greater than eightfold gene expression difference in *Pax5*^∆/∆^ progenitors and *Pax5*^∆CRD1,2/–^ pro-B cells compared with *Pax5*^+/+^ pro-B cells. The indicated activated (green) and repressed (red) genes were additionally selected for the presence of Pax5 binding and/or open chromatin at their promoter regions (A) or putative intragenic enhancers (F) in at least one of the *Pax5*^+/+^, *Pax5*^∆CRD1/∆CRD1^, or *Pax5*^∆CRD1,2/∆CRD1,2^ pro-B cells. The promoter region ranged from −2.5 kb to +1 kb relative to the TSS. **(B and G)** Pax5 binding at the selected promoter regions (B) or putative intragenic enhancers (G) of activated, repressed, or non-regulated genes in *Pax5*^+/+^, *Pax5*^∆CRD1/∆CRD1^, and *Pax5*^∆CRD1,2/∆CRD1,2^ pro-B cells. The horizontal lines of the box plots indicate the median density of Pax5 binding, while the boxes represent the middle 50% of the data and the whiskers denote all values of the 1.5× interquartile range. Pax5 binding was identified by ChIP-seq analysis of the indicated pro-B cell types with an anti-Pax5 paired domain antibody (Adams et al., 1992; see Materials and methods). The non-regulated genes corresponded to 70 randomly selected genes, which had a log_2_ fold expression change between −0.1 and 0.1 in all pro-B comparisons and exhibited Pax5 binding and/or open chromatin at their promoter regions (B) or putative intragenic enhancers (G). **(C and H)** Open chromatin at the selected promoter regions (C) or putative intragenic enhancers (H) of Pax5-activated, Pax5-repressed, or non-regulated genes in *Pax5*^+/+^, *Pax5*^∆CRD1/∆CRD1^, *Pax5*^∆CRD1,2/∆CRD1,2^ pro-B cells, and *Pax5*^∆/∆^ progenitors. The density of open chromatin, which was determined by ATAC-seq analysis of the indicated pro-B cell types, is presented by box plots as described in B and G. Statistical data (B, C, G, and H) were analyzed by the Mann–Whitney test; *P < 0.05, **P < 0.01, ***P < 0.001, ****P < 0.0001. **(D, E, I, and J)** Pax5 binding and open chromatin at the promoters (red) or putative intragenic enhancers (red) of two activated (D and I) and two repressed (E and J) genes are shown as RPM values for pro-B cells of the indicated genotypes (left). The expression of the corresponding genes in the different pro-B cell types is indicated as mean TPM values of two or five RNA-seq experiments per genotype (right).

Genes in the clusters B, C, and D showed varying dependencies on CRD1 and CRD2 for their activation or repression in pro-B cells. The activation of genes in cluster B was only minimally affected by deletion of CRD2 in *Pax5*^∆CRD2/−^ pro-B cells and was further reduced upon deletion of CRD1 in *Pax5*^∆CRD1/−^ pro-B cells but was largely lost only upon elimination of both C-terminal regulatory domains in *Pax5*^∆CRD1,2/−^ pro-B cells, as exemplified by the genes *Cd19*, *Slamf7*, *Nedd9*, *Bcl2l1* (Bcl-X_L_), *Asb2*, *Arntl1* (Bmal1), *Tcf3* (E2A), and *Lef1* (Fig. 5 C, Fig. 6 D, and Fig. S4 C). Activation of the genes in cluster C totally depended on CRD1 in *Pax5*^∆CRD1/−^ pro-B cells with minimal contribution of CRD2 in *Pax5*^∆CRD2/−^ pro-B cells, as illustrated by the genes *Fcrla*, *Cacna1e*, *Dkk3*, *Tmprss3*, *Lcp2* (Slp76), *Bfsp2*, *Mgst1*, *Plxdc1*, and *Heyl* (Fig. 5 C, Fig. 6 I, and Fig. S4 C). Finally, CRD1 and CRD2 were both similarly important for the activation of genes in cluster D because their expression was lost in *Pax5*^∆CRD1/−^ and *Pax5*^∆CRD2/−^ pro-B cells, as exemplified by the genes *Enpep* (BP-1), *Tmem132e*, *Slc52a3*, *Cldn4*, and *Grb7* (Fig. 5 C and Fig. S4 C).

Upregulation of the repressed genes in cluster B was almost exclusively observed upon deletion of both CRD1 and CRD2 in *Pax5*^∆CRD1,2/−^ pro-B cells with little or no contribution of CRD1 or CRD2 deletion in *Pax5*^∆CRD1/−^ and *Pax5*^∆CRD2/−^ pro-B cells, respectively, as exemplified by the genes *Slc9a2*, *Il18rap*, *Ifitm1*, *Lat2*, *Grap2*, *Ahnak*, *Fut7*, *Sulf2*, and *Lsr* (Fig. 5 D, Fig. 6 J, Fig. 8 G, and Fig. S4 D). Notably, derepression of these genes in *Pax5*^∆CRD1,2/−^ pro-B cells often did not reach the level of expression observed in *Pax5*^∆/∆^ progenitors (Fig. 5 D, Fig. 6 J, Fig. 8 G, and Fig. S4 D). Repression of the genes in cluster C was almost exclusively dependent on CRD1 as these genes were derepressed in *Pax5*^∆CRD1/−^ and *Pax5*^∆CRD1,2/−^ pro-B cells, as manifested by the genes *Ly6a* (Sca1), *Sell* (CD62L), *Trem1*, *Sema3g*, *Gimap4*, *Mylk*, *Pnp2*, and *Car3* (Fig. 5 D, Fig. 6 E, and Fig. S4 D). Lastly, CRD1 and CRD2 were both equally important for repression of the few genes in cluster D, which were derepressed in both *Pax5*^∆CRD1/−^ and *Pax5*^∆CRD2/−^ pro-B cells, as illustrated by the genes *Cgas*, *Eef1akmt1*, *Hnrnpll*, and *Ell3* (Fig. 5 D and Fig. S4 D).

Interestingly, repression of the three genes *Cgas*, *Eef1akmt1*, and *Hnrnpll* in cluster D depended not only on both CRD1 and CRD2 domains but also on the OP, as they were equally derepressed in *Pax5*^∆CRD1/−^, *Pax5*^∆CRD2/−^, and *Pax5*^∆OP/∆OP^ pro-B cells (Figs. 2 G and 5 D; and Figs. S2, A and C; and S4 D). Notably, 11 activated genes in cluster C required not only the CRD1 domain but also the HD for their expression, which was strongly reduced in *Pax5*^∆CRD1/−^ and *Pax5*^∆HD/∆HD^ pro-B cells (Fig. 2 G; and Figs. S2, A and B; and S4 C). Furthermore, 10 genes in cluster D required CRD1, CRD2, as well as the HD for their activation as their expression was greatly decreased or lost in *Pax5*^∆CRD1/−^, *Pax5*^∆CRD2/−^, and *Pax5*^∆HD/∆HD^ pro-B cells (Figs. 2 G and 5 C; and Figs. S2, A and B; and S4 C). Together, these data indicate that a large part (21 [66%]) of the 31 genes, which are activated in an HD-dependent manner, additionally require one or both CRD elements for their expression.

**Figure S5.**
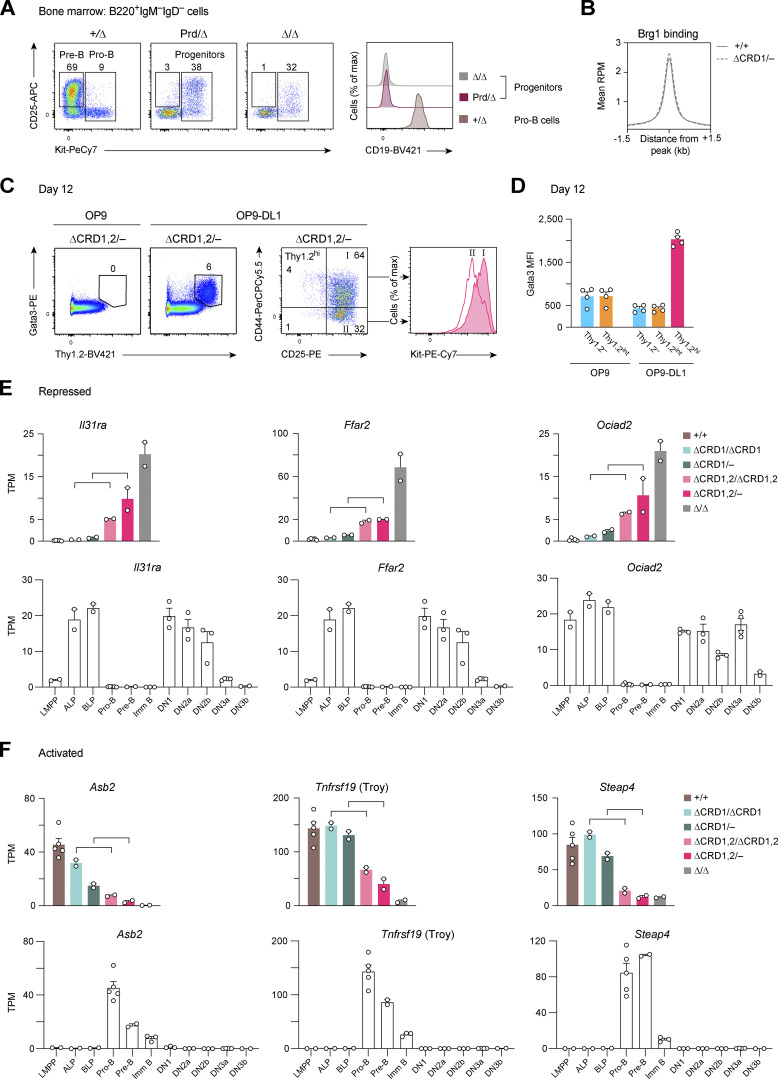
**Expression profiles of Pax5-regulated genes with a potential role in B-lineage commitment. (A)** B cell developmental arrest at an early progenitor cell stage in *Pax5*^Prd/∆^ mice, as shown by flow-cytometric analysis of bone marrow cells from *Pax5*^+/∆^ (*Vav*-Cre *Pax5*^+/fl^), *Pax5*^Prd/∆^ (*Vav*-Cre *Pax5*^Prd/fl^), and *Pax5*^∆/∆^ (*Vav*-Cre *Pax5*^fl/fl^) mice at the age of 6 wk. **(B)** Density profiles of genome-wide Brg1 binding in *Pax5*^+/+^ and *Pax5*^∆CRD1/−^ pro-B cells. The profiles are shown for a region from −1.5 to +1.5 kb relative to the Brg1 peak summit. **(C)** In vitro lymphoid differentiation of cultured *Pax5*^∆CRD1,2/−^ pro-B cells that were seeded on OP9-DL1 feeder cells (Schmitt and Zúñiga-Pflücker, 2002) in IL-7– and Flt3L-containing medium. *Pax5*^∆CRD1,2/−^ pro-B cells, which were further cultured on OP9 feeder cells in IL-7–containing medium, served as a control. After 12 d of co-culture, live lymphoid cells were analyzed by flow cytometry for cell surface Thy1.2 and intracellular Gata3 expression (left). CD44 and CD25 expression on gated Thy1.2^hi^ cells is additionally displayed together with Kit expression on fraction I and II cells (right). The percentage of cells in the indicated gates is shown. **(D)** Gata3 expression is shown as MFI value in the indicated Thy1.2^−^, Thy1.2^int^, or Thy1.2^hi^ cells after 12 d of co-culture. One of two independent experiments (C and D) is shown. **(E and F)** The Pax5-repressed genes *Il31ra*, *Ffar2*, and *Ociad2* (E) and Pax5-activated genes *Asb*, *Tnfrsf19* (Troy), and *Steap* (F) were identified as significantly differentially expressed genes in the comparisons of committed *Pax5*^∆CRD1/∆CRD1^ and *Pax5*^∆CRD1/−^ pro-B cells with uncommitted *Pax5*^∆CRD1,2/∆CRD1,2^ and *Pax5*^∆CRD1,2/−^ pro-B cells, respectively (Fig. 8 F). Top rows: The expression of the three Pax5-repressed (E) and three Pax5-activated (F) genes in pro-B and progenitor cells of the indicated genotypes is shown as the mean TPM value of two or five RNA-seq experiments per genotype. Brackets indicate the two comparisons used for the identification of these genes. Bottom rows: The expression of the three Pax5-repressed (E) and three Pax5-activated (F) genes is shown as mean TPM values for wild-type lymphoid progenitors (LMPP, ALP, BLP; two experiments) and early B cells (pro-B, pre-B, and immature (Imm) B cells; two to five experiments) from the bone marrow as well as for early T cell progenitors (ETP/DN1, DN2a, DN2b, DN3a, and DN3b; two to four experiments) from the thymus. The definition of the different lymphoid progenitors and T cell precursors is described in Materials and methods. DN, CD4^−^CD8^−^ double-negative thymocytes.

The largest cluster A of the heatmap contains 216 (38%) of all 567 activated genes and 326 (56%) of all 580 repressed genes (Fig. 5 B), whose differential expression reached significance only in the comparison between *Pax5*^∆/∆^ progenitors and *Pax5*^+/+^ pro-B cells. Hence, deletion of CRD1, CRD2, or both domains minimally affected the activation of genes in cluster A in *Pax5*^∆CRD1/−^, *Pax5*^∆CRD2/−^, or *Pax5*^∆CRD1,2/−^ pro-B cells, as exemplified by the genes *Vpreb1*, *Vpreb3*, *Cd79a* (Igα), *Scn4a*, *Sit1*, *Pclaf*, *Ndc80*, and *Kif11* (Fig. 5, C and E; and Fig. S4 C). Likewise, genes that were expressed in *Pax5*^∆/∆^ progenitors were not derepressed upon deletion of CRD1, CRD2, or both domains in the respective pro-B cells, as manifested by the genes *Itgax*, *Cd7*, *Cd27*, *Fyb*, *Htra3*, *Ptpn3*, *Orm2*, *Chdh*, and *Nrgn* (Fig. 5 D and Fig. S4 D). At face value, these data could indicate that most genes in cluster A are not at all or only minimally regulated by Pax5 in pro-B cells. While this statement may be true for many genes in cluster A, there are some notable exceptions. For instance, the genes *Ptpn3*, *Orm2*, and *Chdh* are derepressed in *Pax5*^∆OP/∆OP^ pro-B cells, indicating that the OP of Pax5 mediates the repression of these three genes in pro-B cells instead of the C-terminal domains (Fig. S2, A and D). Moreover, Pax5 was previously implicated in the activation of the *Cd79a* gene by recruiting members of the Ets transcription factor family via its paired domain to the *Cd79a* promoter (Fitzsimmons et al., 1996; Garvie et al., 2001; Nutt et al., 1998). We therefore next investigated whether the paired domain (Prd) plays a similar role in the activation of other cluster A genes in addition to *Cd79a* by taking advantage of the *Pax5*^Prd^ allele expressing only Prd from the *Pax5* locus (Smeenk et al., 2017). B cell development was stringently arrested at a similar CD19^−^B220^+^Kit^+^ progenitor cell stage in *Pax5*^Prd/∆^ (*Vav*-Cre *Pax5*^Prd/fl^) and *Pax5*^∆/∆^ mice (Fig. S5 A). By RNA-seq analysis, we identified a relatively small number of genes that was differentially expressed between *Pax5*^Prd/∆^ and *Pax5*^∆/∆^ progenitors (Table S3). By additionally considering Prd binding based on chromatin immunoprecipitation (ChIP) coupled with deep sequencing (ChIP-seq) analysis of *Pax5*^Prd/∆^ progenitors, we identified only two cluster A genes, the previously known *Cd79a* and the newly found *Vpreb3*, that were both activated and bound by the Pax5 paired domain in *Pax5*^Prd/∆^ progenitors (Fig. 5 E). In summary, these data do not support a role of the five analyzed Pax5 domains in the regulation of most cluster A genes, although Pax5 binding was observed at 84% of the activated cluster A genes and 53% of the repressed cluster A genes in *Pax5*^+/+^ pro-B cells (Table S2).

### Control of chromatin accessibility and Pax5 binding by the C-terminal domains of Pax5

We next investigated whether the C-terminal regulatory domains are involved in the control of open chromatin or Pax5 binding at Pax5-regulated genes. For this purpose, we performed ChIP-seq analysis with an anti-Pax5 paired domain antibody (Adams et al., 1992) to determine Pax5-binding in *Pax5*^+/+^, *Pax5*^∆CRD1/∆CRD1^, and *Pax5*^∆CRD1,2/∆CRD1,2^ pro-B cells. Moreover, we mapped open chromatin in *Pax5*^+/+^, *Pax5*^∆CRD1/∆CRD1^, *Pax5*^∆CRD1,2/∆CRD1,2^ pro-B cells, and *Pax5*^∆/∆^ progenitors by ATAC-seq analysis (Buenrostro et al., 2013). We then selected genes that were greater than eightfold deregulated in *Pax5*^∆CRD1,2/−^ pro-B cells and *Pax5*^∆/∆^ progenitors relative to *Pax5*^+/+^ pro-B cells and that additionally exhibited Pax5 binding and/or open chromatin at their promoters (Fig. 6 A) or intragenic enhancers (Fig. 6 F) in one of the three pro-B cell types. Furthermore, 70 randomly selected, non-regulated genes were used as control to demonstrate that Pax5 binding and open chromatin did not change significantly at their promoters or intragenic enhancers in the different pro-B cell types (Fig. 6, B, C, G, and H). In contrast, Pax5 binding and open chromatin at the promoter region or intragenic enhancers of activated genes was gradually decreased from *Pax5*^+/+^ pro-B cells to *Pax5*^∆CRD1/∆CRD1^ and *Pax5*^∆CRD1,2/∆CRD1,2^ pro-B cells (Fig. 6, B, C, G, and H), as exemplified for the promoter regions of *Fxyd6* and *Cd19* (Fig. 6 D) and intragenic enhancers of *Id3* and *Plxdc1* (Fig. 6 I). An inverse picture was observed for gene repression by the C-terminal domains. Pax5 binding and open chromatin at the promoters or intragenic enhancers of repressed genes was progressively increased from *Pax5*^+/+^ pro-B cells to *Pax5*^∆CRD1/∆CRD1^ and *Pax5*^∆CRD1,2/∆CRD1,2^ pro-B cells (Fig. 6, B, C, G, and H), which is highlighted for the promoter regions of *Ehd3* and *Trem1* (Fig. 6 E) and intragenic enhancers of *Flt3* and *Lsr* (Fig. 6 J). In summary, these data demonstrate that the C-terminal regulatory domains of Pax5 contribute to the control of open chromatin as well as Pax5 binding at regulated genes.

### The CRD1 domain of Pax5 interacts with several coactivator and corepressor complexes

**Figure 7.**
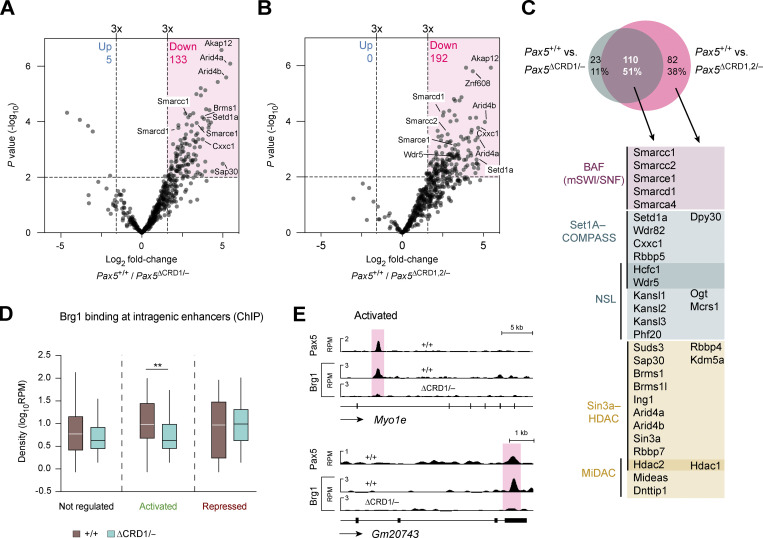
**Identification of protein complexes interacting with the CRD1 domains of Pax5. (A and B)** Volcano plots displaying the preferential association of proteins with Pax5 in nuclear extracts of *Pax5*^+/+^ pro-B cells compared with *Pax5*^∆CRD1/−^ (A) or *Pax5*^∆CRD1,2/−^ (B) pro-B cells. Proteins were identified as specifically associated with the CRD domain if they exhibited a difference in abundance of greater than threefold with a P value of <0.01 in the Co-IP–MS experiments of *Pax5*^+/+^ versus mutant pro-B cells (Table S4). Two independent Co-IP–MS experiments were performed each with six replicates for *Pax5*^∆CRD1/–^ and *Pax5*^∆CRD1,2/−^ pro-B cells and three replicates for *Pax5*^+/+^ pro-B cells. **(C)** Pax5-interacting proteins identified by the different pro-B cell comparisons shown in A and B. The overlapping proteins, which depend on the CRD1 domain for their interaction with Pax5, contained the indicated components of the chromatin-remodeling complex BAF (Hodges et al., 2016) and the four histone-modifying complexes Set1A-COMPASS (Cenik and Shilatifard, 2021), NSL (Sheikh et al., 2019), Sin3-HDAC (Bansal et al., 2016), and MiDAC (Turnbull et al., 2020). Synonymous names of some components are Smarca4 (Brg1), Smarcc1 (Baf155), Smarcc1 (Baf170), Smarcd1 (Baf60A), Smarce1 (Baf57), Setd1a (Set1a), Arid4a (Rbp1), Arid4b (Sap180), and Suds3 (Sds3). **(D)** Brg1 binding at putative intragenic enhancers of Pax5-activated, Pax5-repressed, or non-regulated genes, as determined by ChIP-seq analysis of in vitro cultured *Pax5*^+/+^ and *Pax5*^∆CRD1/−^ pro-B cells with an anti-Brg1 (Smarca4) antibody (Wang et al., 2020). The horizontal lines of the box plots indicate the median density of Brg1 binding, while the boxes represent the middle 50% of the data and the whiskers denote all values of the 1.5× interquartile range. Brg1 binding was analyzed at intragenic enhancers of genes with an greater than eightfold activation or repression by Pax5, as defined in Fig. 6 F. Intragenic enhancers of non-regulated genes, which were identified as described in the legend of Fig. 6 G, were used as control. Statistical data were analyzed by the Mann–Whitney test; **P < 0.01. **(E)** Binding of Brg1 and Pax5 at putative intragenic enhancers (red) of the Pax5-activated genes Myo1e and Gm20743 are shown as RPM values for *Pax5*^+/+^ and *Pax5*^∆CRD1/−^ pro-B cells.

We next performed co-immunoprecipitation (Co-IP) experiments combined with mass spectrometric analysis (MS) to identify coactivator or corepressor complexes that bind to the C-terminal region of Pax5. To this end, we cultured *Pax5*^∆CRD1/−^, *Pax5*^∆CRD1,2/−^, and *Pax5*^+/+^ pro-B cells prior to nuclear extract preparation and Co-IP with an anti-Pax5 paired domain antibody (Adams et al., 1992). We performed two experiments each with *Pax5*^∆CRD1/−^ and *Pax5*^+/+^ pro-B cells (Fig. 7 A) as well as with *Pax5*^∆CRD1,2/−^ and *Pax5*^+/+^ pro-B cells (Fig. 7 B). Proteins were identified as specifically associated with a CRD domain if they exhibited a difference in abundance of greater than threefold with a P value of <0.01 in the Co-IP–MS experiments of wild-type versus mutant pro-B cells (Table S4). The comparison between *Pax5*^∆CRD1/−^ and *Pax5*^+/+^ pro-B cells or *Pax5*^∆CRD1,2/−^ and *Pax5*^+/+^ pro-B cells identified 133 or 192 proteins, respectively, that were associated at a significantly reduced frequency with Pax5-∆CRD1 or Pax5-∆CRD1,2 compared with full-length Pax5 protein (Fig. 7, A and B; and Table S4). The overlap of both analyses resulted in 110 proteins that significantly interact with the CRD1 domain of Pax5 (Fig. 7 C). These shared proteins contain five members of the chromatin-remodeling BAF (mSWI/SNF) complex (Hodges et al., 2016), six components of the H3K4-methylating Set1A-COMPASS complex (Cenik and Shilatifard, 2021), six members of the H4K16-acetylating NSL complex (Sheikh et al., 2019), ten components of the Sin3-HDAC corepressor complex (Bansal et al., 2016), and three proteins of the MiDAC histone deacetylase complex (Turnbull et al., 2020; Fig. 7 C). Moreover, few additional components of these complexes were identified with stringent cutoffs only in the Co-IP–MS comparison between *Pax5*^∆CRD1,2/−^ and *Pax5*^+/+^ pro-B cells (Fig. 7 C). Together, these data suggest that the CRD1 domain of Pax5 can promote transcriptional activation by associating with the BAF, Set1A-COMPASS, or NLS complexes, while transcriptional repression may be mediated by its interaction with the Sin3-HDAC or MiDAC complexes.

To investigate whether the CRD1 domain is able to recruit these coactivator or corepressor complexes to Pax5-binding sites of regulated genes, we investigated binding of the BAF complex in *Pax5*^+/+^ and *Pax5*^∆CRD1/−^ pro-B cells by ChIP-seq analysis with an antibody directed against the BAF ATPase Brg1 (Smarca4; Wang et al., 2020). While genome-wide binding of Brg1 was similar in both pro-B cell types (Fig. S5 B), the analysis of intragenic enhancers of genes with a greater than eightfold regulation by Pax5 (Fig. 6 F) revealed a significant reduction of Brg1 binding at enhancers of activated genes in *Pax5*^∆CRD1/−^ pro-B cells compared with *Pax5*^+/+^ pro-B cells (Fig. 7, D and E). In contrast, Brg1 binding was similar at intragenic enhancers of repressed or non-regulated genes (Fig. 7 D). We conclude therefore that the CRD1 domain is responsible for recruiting the chromatin-remodeling BAF complex to Pax5-binding sites at intragenic enhancers of activated Pax5 target genes.

### Loss of B cell commitment upon deletion of both C-terminal domains of Pax5

**Figure 8.**
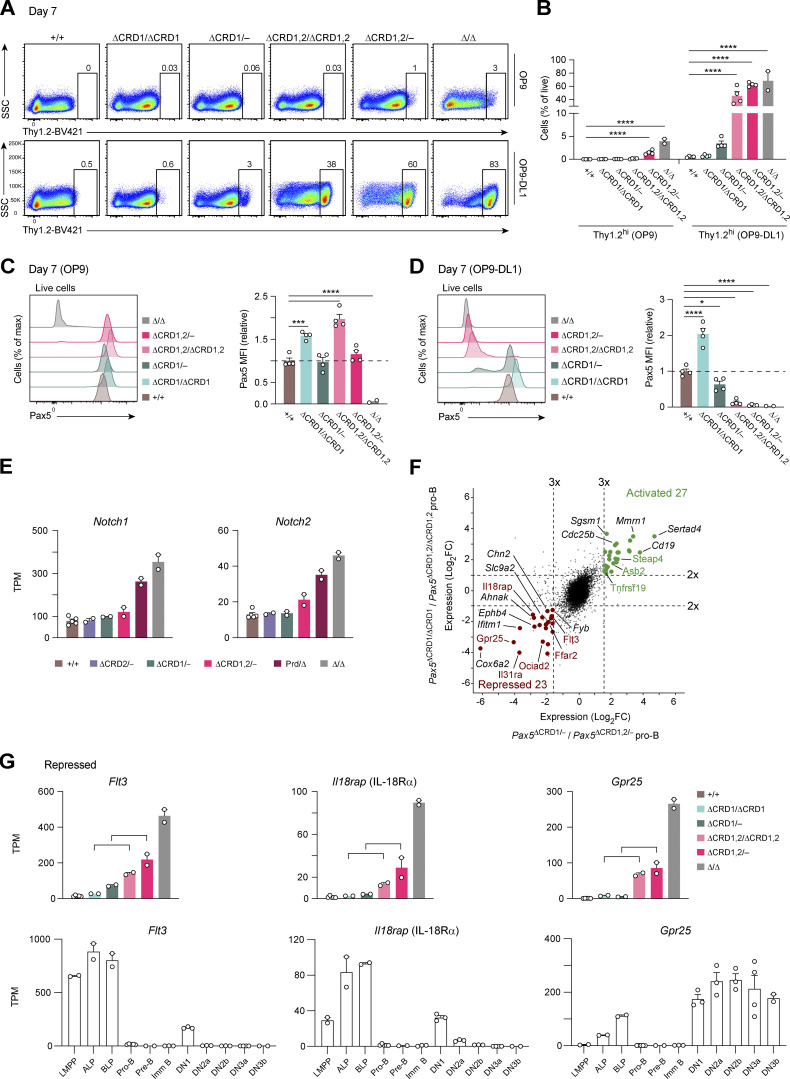
**Critical role of the C-terminal Pax5 domains in regulating B cell commitment. (A)** In vitro T cell differentiation of pro-B cells of the indicated genotypes and the control *Pax5*^∆/∆^ progenitors. Ex vivo sorted pro-B cells were short-term cultured on OP9 feeder cells in IL-7–containing medium prior to seeding half of the pro-B cells on OP9-DL1 feeder cells (Schmitt and Zúñiga-Pflücker, 2002) in IL-7– and Flt3L-containing medium and the other half of pro-B cells on OP9 cells in IL-7–containing medium. After 7 d of co-culture, live lymphoid cells were analyzed by flow cytometry. Numbers refer to the percentage of Thy1.2^hi^ cells in the indicated gate. One of two independent experiments is shown. **(B)** Frequency of Thy1.2^hi^ cells after 7 d of co-culture of the indicated pro-B cells with OP9 or OP-DL1 cells, shown as mean values with SEM (*n* ≥ 2). **(C and D)** Pax5 expression in pro-B cells of the indicated genotypes after 7 d of co-culture with OP9 cells in IL-7–containing medium (C) or with OP9-DL1 cells in IL-7– and Flt3L-containing medium (D), as determined by flow-cytometric analysis of intracellular Pax5 staining (left). The geometric MFI of the different pro-B cells is displayed relative to that of control *Pax5*^+/+^ pro-B cells (set to 1; right) and is shown as mean values with SEM (*n* ≥ 2). Statistical data (B–D) were analyzed by one-way ANOVA with Dunnett’s multiple comparison test; *P < 0.05, ***P < 0.001, ****P < 0.0001. Each dot (B–D) corresponds to one mouse. One of two independent experiments (A–D) is shown. **(E)** Expression of *Notch1* and *Notch2* (cluster A genes) in pro-B and progenitor cells of the indicated genotypes is shown as mean TPM values of two or five RNA-seq experiments per genotype. **(F)** Overlap of Pax5-activated and Pax5-repressed genes that are differentially expressed both in the comparison between committed *Pax5*^∆CRD1/∆CRD1^ and uncommitted *Pax5*^∆CRD1,2/∆CRD1,2^ pro-B cells as well as in the comparison between committed *Pax5*^∆CRD1/−^ and uncommitted *Pax5*^∆CRD1,2/−^ pro-B cells (Table S5). Genes with an adjusted P value of <0.05, a mean TPM value of >5 in one pro-B cell type and an expression difference of greater than threefold (between *Pax5*^∆CRD1/−^ and *Pax5*^∆CRD1,2/−^ cells) or greater than twofold (between *Pax5*^∆CRD1/∆CRD1^ and *Pax5*^∆CRD1,2/∆CRD1,2^ cells) are colored in green or red, corresponding to commonly activated or repressed genes, respectively. **(G)** Top row: Expression of the Pax5-repressed genes *Flt3*, *Il18rap*, and *Gpr25* in pro-B and progenitor cells of the indicated genotypes is shown as mean TPM values of two or five RNA-seq experiments per genotype. Brackets indicate the two comparisons between committed pro-B cells (*Pax5*^∆CRD1/∆CRD1^ and *Pax5*^∆CRD1/−^) and uncommitted pro-B cells (*Pax5*^∆CRD1,2/∆CRD1,2^ and *Pax5*^∆CRD1,2/−^) used for identification of these genes. Bottom row: The expression of *Flt3*, *Il18rap*, and *Gpr25* is shown as mean TPM values in lymphoid progenitors (LMPP, ALP, BLP; two experiments) and early B cells (pro-B, pre-B, and immature [imm] B cells; two to five experiments) from the bone marrow as well as in early T cell precursors (ETP/DN1, DN2a, DN2b, DN3a, and DN3b; two to four experiments) from the thymus. The definition of the different lymphoid progenitors and T cell precursors is described in Materials and methods. DN, CD4^–^CD8^–^ double-negative thymocytes.

We next investigated a potential role of the C-terminal domains of Pax5 in the control of B-lineage commitment. For this purpose, we used an in vitro differentiation system, which is based on the induction of Notch signaling in lymphoid progenitors that are cultured on OP9-Delta-like 1 (OP9-DL1) feeder cells expressing the Delta-like Notch ligand 1 (Schmitt and Zúñiga-Pflücker, 2002). Using this differentiation system, we have previously shown that uncommitted *Pax5*^−/−^ progenitors efficiently differentiate to Thy1.2^hi^ T-lymphoid precursor cells within 7 d of co-culture on OP9-DL1 cells (Höflinger et al., 2004). To investigate the differentiation potential, we short-term cultured ex vivo sorted pro-B cells from *Pax5*^+/+^, *Pax5*^∆CRD1/∆CRD1^, *Pax5*^∆CRD1/−^, *Pax5*^∆CRD1,2/∆CRD1,2^, *Pax5*^∆CRD1,2/−^, and control *Pax5*^∆/∆^ mice on OP9 cells in IL-7–containing medium prior to seeding half of the pro-B cells on OP9 cells (plus IL-7) and the other half of the pro-B cells on OP9-DL1 cells (plus IL-7 and Flt3L). After 7 d in culture, the cells of the different genotypes maintained their pro-B cells phenotype on OP9 cells, as shown by their Pax5 expression except for the *Pax5*^∆/∆^ progenitors (Fig. 8, A–C). While the *Pax5*^+/+^, *Pax5*^∆CRD1/∆CRD1^, and *Pax5*^∆CRD1/−^ pro-B cells maintained their committed B cell phenotype upon co-culture on OP9-DL1 cells, as shown by their continued expression of Pax5 (Fig. 8 D), the *Pax5*^∆CRD1,2/∆CRD1,2^ and *Pax5*^∆CRD1,2/−^ pro-B cells as well as *Pax5*^∆/∆^ progenitors differentiated on OP9-DL1 cells into Thy1.2^hi^ cells (Fig. 8, A and B) that lost Pax5 expression (Fig. 8 D) and gained Gata3 expression (Fig. S5, C and D). Upon further analysis, the *Pax5*^∆CRD1,2/−^ Thy1.2^hi^ cells could be divided into a larger (64%) fraction I of CD44^+^CD25^hi^Kit^hi^ cells and a smaller (32%) fraction II of CD44^−^CD25^hi^Kit^int^ cells, which could correspond to DN2-like cells (Thy1.2^hi^Gata3^+^CD44^+^CD25^hi^Kit^hi^) or DN3-like cells (Thy1.2^hi^Gata3^+^CD44^−^CD25^hi^Kit^int^), respectively (Yui et al., 2010; Fig. S5 C). In the absence of further characterization, it is also possible that the fraction I and II cells may be ILC2 or ILC3 cells. Irrespective of what cell type has been generated, this differentiation experiment demonstrated that only the *Pax5*^∆CRD1,2/∆CRD1,2^ and *Pax5*^∆CRD1,2/−^ pro-B cells could undergo differentiation along another lineage. We conclude therefore that the CRD1 and CRD2 domains together determine the B-lineage commitment function of Pax5.

We next attempted to identify Pax5-regulated genes that may be involved in B-lineage commitment. As Pax5 has been implicated in the fivefold repression of the T cell fate specification gene *Notch1* (Radtke et al., 1999) in committed pro-B cells (Souabni et al., 2002), we first analyzed the expression of *Notch1* in *Pax5*^+/+^, *Pax5*^∆CRD2/−^, *Pax5*^∆CRD1/−^, and *Pax5*^∆CRD1,2/−^ pro-B cells as well as in *Pax5*^Prd/∆^ and *Pax5*^∆/∆^ progenitor cells (Fig. 8 E). Surprisingly, *Notch1* and *Notch2* did not depend on the CRD1 and CRD2 domains of Pax5 for their repression in pro-B cells, while both genes were also not repressed upon expression of the Pax5 paired domain in *Pax5*^Prd/∆^ progenitors (Fig. 8 E). As the two *Notch* genes were also not derepressed upon loss of the OP or HD in *Pax5*^∆OP/∆OP^ or *Pax5*^∆HD/∆HD^ pro-B cells, respectively (Table S1), we conclude that none of the Pax5 domains is involved in *Notch* gene repression. These data strongly suggest that the cluster A genes *Notch1* and *Notch2* are also repressed in a Pax5-independent manner during the transition from uncommitted progenitors to committed pro-B cells.

To systematically search for Pax5-dependent genes that may contribute to B-lineage commitment, we first analyzed ex vivo sorted *Pax5*^∆CRD1/∆CRD1^ and *Pax5*^∆CRD1,2/∆CRD1,2^ pro-B cells by RNA-seq and then identified genes that were differentially expressed between committed *Pax5*^∆CRD1/∆CRD1^ and uncommitted *Pax5*^∆CRD1,2/∆CRD1,2^ pro-B cells or between committed *Pax5*^∆CRD1/−^ and uncommitted *Pax5*^∆CRD1,2/−^ pro-B cells (Table S5). By overlapping the two datasets, we identified 23 Pax5-repressed and 27 Pax5-activated genes that exhibited a significant difference in expression between the committed and uncommitted pro-B cells (Fig. 8 F). The expression patterns of six Pax5-repressed genes (*Flt3*, *Il18rap*, *Gpr25*, *Il31ra*, *Ffar2*, and *Ociad2*) and three Pax5-activated genes (*Asb2*, *Tnfrsf19*, and *Steap4*) are shown for wild-type and *Pax5* mutant pro-B cells as well as for uncommitted lymphoid progenitors (lymphoid-primed multipotent progenitors [LMPP], all-lymphoid progenitors [ALP], and B cell–biased lymphoid progenitors [BLP]) and early B cell types (pro-B, pre-B, and immature B cells) in the bone marrow and for early T-lymphoid precursor cells (early T cell progenitors [ETP]/DN1, DN2a, DN2b, DN3a, and DN3B cells) in the thymus (Fig. 8 G and Fig. S5, E and F). While the three Pax5-activated genes were exclusively expressed in early B cell development, the six Pax5-repressed genes were expressed in lymphoid progenitors and thymic T-lymphoid precursor cells but not in early B cells, consistent with a possible function of some of these Pax5-repressed genes in early lymphopoiesis and/or early T cell development. Five of the Pax5-repressed genes code for cell surface proteins (Flt3, Il18rap [IL-18Rβ], Il31ra [IL-31Rα], Gpr25, and Ffar2). Notably, the *Il18r1* gene encoding the IL-18Rα chain is also a Pax5-repressed gene and exhibited a similar expression pattern as *Il18rap* (IL-18Rβ; Table S2), which points to the presence of a functional IL-18R on early lymphoid progenitors and the earliest DN1 progenitor cells. As the repression of *Flt3* was previously shown to be crucial for B-lineage commitment (Holmes et al., 2006), it is conceivable that the downregulated expression of some of the other 22 Pax5-repressed genes may also contribute to the generation of committed pro-B cells.

## Discussion

Pax5 is a key regulator of B cell immunity that controls B-lineage commitment (Nutt et al., 1999), V_H_-DJ_H_ recombination of the *Igh* locus (Hill et al., 2020), and the development of all mature B cell types (Calderón et al., 2021). *PAX5* also plays an important role as a tumor suppressor gene in B-ALL development (Gu et al., 2019; Mullighan et al., 2007). The Pax5 protein consists of the N-terminal paired domain, a conserved OP, HD, and C-terminal sequences (Bouchard et al., 2008). These conserved domains are differently affected by *PAX5* mutations in human B-ALL as missense mutations cluster in the paired domain and frameshift mutations are prevalent in the C-terminal domain (Gu et al., 2019). Moreover, the germline mutation G183S in the OP confers inherited susceptibility to B-ALL development (Auer et al., 2014; Shah et al., 2013), while the HD is largely devoid of gene mutations in B-ALL (Gu et al., 2019). Here, we determined the in vivo function of the OP, HD, and C-terminal sequences by deletion in the *Pax5* gene. We previously analyzed the function of the Pax5 C-terminal domain by mutagenesis and expression in established B cell lines. These transient transfection experiments, which were performed with artificial reporter genes containing three copies of a high-affinity Pax5-binding site, identified a potent TAD and an adjacent ID (Dörfler and Busslinger, 1996). Here, we have shown by RNA-seq analysis of pro-B cells lacking the CRD1 or CRD2 domain that CRD1, encompassing the TAD, and CRD2, containing two-thirds of the ID sequences, each contribute to both activation and repression of Pax5-regulated gene. Hence, the previously identified separable transactivation and inhibitory functions of the C-terminal sequences are likely a consequence of the artificial design of the transient transfection experiments (Dörfler and Busslinger, 1996).

Deletion of the Pax5 OP or HD had minor effects on B lymphopoiesis, although both sequences together were required for optimal B cell development. Interestingly, the two sequence motifs have opposing transcriptional functions, as the OP is primarily involved in gene repression, while the HD largely contributes to gene activation. The repression function of the OP is consistent with our previous finding that members of the Groucho (Grg/Tle) corepressor family can specifically interact with the OP sequence of Pax5 (Eberhard et al., 2000). This previous analysis also revealed that the central SP and N-terminal Q domains of Grg4 (Tle4) interact with Pax5 by binding to the OP and the TAD (CRD1), respectively (Eberhard et al., 2000). This two-pronged mode of binding could explain why the three genes *Cgas*, *Eef1akmt1*, and *Hnrnpll* require both the OP and CRD1 domain for their repression.

Consistent with the activation function of the HD, we previously demonstrated by biochemical analysis that the TATA-binding protein of the transcription initiation complex TFIID can specifically interact with the HD of Pax5 (Eberhard and Busslinger, 1999). Moreover, pulldown experiments with in vivo biotinylated Pax5 protein combined with MS analysis previously identified several components of the TFIID complex including TATA-binding protein, which demonstrated that the full-length Pax5 protein can interact with the basal transcription machinery in pro-B cells (McManus et al., 2011). Our current Co-IP–MS analyses did not, however, identify binding of any TFIID components to the CRD1 and CRD2 domains of Pax5, thus supporting the previous finding that TFIID interacts with the HD. Interestingly however, 66% (21) of the 32 HD-dependent genes also require the CRD1 domain for their activation, suggesting that the activation of these genes may depend on the cooperative interaction between TFIID, bound to the HD, and a coactivator complex (BAF, Set1A-COMPASS, or NSL), bound to CRD1 of Pax5.

Whereas deletion of CRD2 of Pax5 has only a minor effect on B cell development, elimination of CRD1 arrests B lymphopoiesis at the pro-B cell stage. Consistent with this finding, CRD1 and CRD2 are responsible for the activation or repression of most Pax5-regulated genes, which can be further distinguished according to their differential dependency on CRD1 and CRD2. Pax5 is known to function as an epigenetic regulator by inducing or eliminating open chromatin and active histone marks at Pax5-binding sites of activated or repressed Pax5 target genes, respectively (McManus et al., 2011; Revilla-i-Domingo et al., 2012). Here, we have shown that this epigenetic regulation is largely mediated by the CRD1 and CRD2 domains of Pax5, as open chromatin was progressively lost or gained at Pax5-binding sites in promoters and enhancers of activated or repressed genes from *Pax5*^+/+^ pro-B cells to *Pax5*^∆CRD1/∆CRD1^ and *Pax5*^∆CRD1,2/∆CRD1,2^ pro-B cells, respectively. Notably, the level of Pax5 binding paralleled that of open chromatin at activated and repressed genes, which can be explained in the following way. While binding of the full-length Pax5 protein in *Pax5*^+/+^ pro-B cells likely induces active chromatin by recruiting coactivator complexes to activated genes, Pax5 proteins lacking CRD1 and/or CRD2 are unable to interact with these coactivator complexes and thus cannot generate open chromatin, which interferes with binding of Pax5 to its sites at activated genes in *Pax5*^∆CRD1/∆CRD1^ and *Pax5*^∆CRD1,2/∆CRD1,2^ pro-B cells. Conversely, Pax5 binding at repressed genes was low in *Pax5*^+/+^ pro-B cells, as binding of the full-length Pax5 protein at the onset of pro-B cell differentiation initiates the shutdown of open chromatin, which, upon loss of accessible chromatin, prevents further Pax5 binding. Consistent with this interpretation, Pax5 proteins lacking CRD1 and/or CRD2 can no longer recruit corepressor complexes, are unable to suppress open chromatin, and therefore continue to interact with their binding sites in promoters and enhancers of repressed genes.

Early B cell development depends on the BAF chromatin-remodeling complex (Bossen et al., 2015; Choi et al., 2012), which induces DNA accessibility by locally disrupting nucleosomes (Cenik and Shilatifard, 2021; Hodges et al., 2016) and has previously been shown to be recruited to Pax5 target genes through its interaction with full-length Pax5 protein (McManus et al., 2011). Here, we have demonstrated that the CRD1 domain of Pax5 specifically interacts with the BAF complex and that the loss of CRD1 prevents recruitment of the BAF complex to intragenic enhancers of activated Pax5 target genes, thus leading to loss of open chromatin at these genes in *Pax5*^∆CRD1/∆CRD1^ pro-B cells. CRD1 also interacts with the H3K4-methylating Set1A-COMPASS (Cenik and Shilatifard, 2021), H4K16-acetylating NSL (Sheikh et al., 2019), Sin3-HDAC (Bansal et al., 2016), and MiDAC (Turnbull et al., 2020) complexes. Upon Pax5-dependent recruitment, the Set1A-COMPASS and NSL complexes likely promote an active chromatin state at activated Pax5 target genes, while the Sin3-HDAC and MiDAC corepressor complexes recruit histone deacetylases to Pax5 target genes to induce gene silencing by eliminating the active histone acetylation marks. An interesting but unresolved question is what determines that the CRD1 domain of Pax5 is able to recruit coactivator complexes to activated Pax5 target genes and corepressor complexes to repressed Pax5 target genes. It is possible that Pax5 may require another transcription factor for co-recruitment of the same coactivator or corepressor complex to its target gene. Interestingly, however, the multisubunit coactivator and corepressor complexes often also contain a protein with a histone modification reader or methyl-CpG binding function (Cenik and Shilatifard, 2021; Hodges et al., 2016; Sheikh et al., 2019). It is therefore conceivable that Pax5-mediated recruitment together with sensing of the active or repressive chromatin environment by the cofactor complex may lead to efficient recruitment of cofactors to activated or repressed Pax5 target genes.

We previously identified Pax5-regulated genes by comparing the gene expression pattern between Pax5-deficient progenitors and wild-type pro-B cells (Delogu et al., 2006; Revilla-i-Domingo et al., 2012; Schebesta et al., 2007). As the Pax5-deficient progenitors are arrested in development before the pro-B cell stage, we used Pax5 binding as a further criterion to define activated or repressed Pax5 target genes, although a significant fraction of these genes may still be differentially expressed due to the distinct regulatory environments of the two cell types instead of the presence or absence of Pax5. By analyzing the role of the different Pax5 domains in gene regulation, we now realized that almost half of all Pax5-regulated genes (present in cluster A) are not at all or only minimally deregulated in the absence of both C-terminal domains in contrast to their strong deregulation in Pax5-deficient progenitors. Some of these cluster A genes could be regulated by other Pax5 domains or by the Pax5 sequences present in the interdomain regions that we did not analyze in this study. Notably, the OP of Pax5 mediates the repression of the three cluster A genes *Ptpn3*, *Orm2*, and *Chdh* instead of the C-terminal domains. Moreover, the Pax5 paired domain alone is already sufficient to induce *Cd79a* and *Vpreb3* expression, which is consistent with a previously reported role of the paired domain in the activation of *Cd79a* by recruiting members of the Ets transcription factor family to the *Cd79a* promoter (Fitzsimmons et al., 1996; Garvie et al., 2001; Nutt et al., 1998). Our current data therefore indicate that Pax5 may not at all or only minimally be involved in the regulation of most cluster A genes. Instead, the lack of expression of “activated” genes or the expression of “repressed” genes in Pax5-deficient progenitors may be caused by the distinct regulatory environment of these progenitors due to their early developmental arrest prior to the committed pro-B cell stage (Nutt et al., 1997).

Pax5 is essential for the commitment of lymphoid progenitors to the B cell pathway by repressing B lineage–inappropriate genes and activating B cell–specific genes at the onset of B cell development (Delogu et al., 2006; Nutt et al., 1999; Schebesta et al., 2007). Here, we have shown that the B-lineage commitment function of Pax5 critically depends on both the CRD1 and CRD2 domains. While Pax5 has been implicated in the repression of the T cell fate specification gene *Notch1* in committed pro-B cells (Souabni et al., 2002), we now demonstrate that *Notch1* repression is likely independent of Pax5 in pro-B cells, as it does not involve any of the five Pax5 protein domains analyzed. Systematic screening for genes with differential expression between committed *Pax5*^∆CRD1/∆CRD1^ and *Pax5*^∆CRD1/−^ pro-B cells and uncommitted *Pax5*^∆CRD1,2/∆CRD1,2^ and *Pax5*^∆CRD1,2/−^ pro-B cells identified 23 Pax5-repressed and 27 Pax5-activated genes, some of which may contribute to B-lineage commitment. One of the Pax5-repressed genes codes for the cytokine receptor Flt3 which is essential for the physiological expansion of lymphoid progenitors in the bone marrow (Mackarehtschian et al., 1995) and for the maintenance of early T cell development in the thymus (Kenins et al., 2010). Pax5-mediated repression of *Flt3* was previously shown to be important for B-lineage commitment, as ectopic expression of *Flt3* in hematopoietic progenitors interferes with early B cell development (Holmes et al., 2006). Similar experiments will be required to further investigate whether other genes among the 23 identified Pax5-repressed genes may also need to be downregulated, like *Flt3*, to facilitate B-lineage commitment.

## Materials and methods

### Mice

The following mice were maintained on the C57BL/6 genetic background: *Pax5*^+/−^ mouse (Urbánek et al., 1994), *Pax5*^fl/fl^ mouse (Horcher et al., 2001), *Pax5*^Prd/+^ mouse (Smeenk et al., 2017), *Meox2*^Cre/+^ mouse (Tallquist and Soriano, 2000), and the transgenic *Vav*-Cre mouse (de Boer et al., 2003), which induces Cre-mediated deletion in hematopoietic stem cells and all hematopoietic lineages. All mouse experiments were performed with littermates and were carried out according to valid project licenses, which were approved and regularly controlled by the Austrian Veterinary Authorities.

### Generation of mutant *Pax5* alleles

The *Pax5*^∆OP^ allele was generated by ES cell targeting (Fig. S1, A and B). To this end, the OP was deleted by PCR amplification, and the corresponding PCR fragment was cloned between the SacII and XhoI sites into the targeting vector containing 1.8- and 4.5-kb-long homology regions and a *loxP*-flanked HSV thymidine kinase promoter-driven neomycin resistance gene. Following blastocyst injection of correctly targeted ES cells and subsequent germline transmission, the *loxP*-flanked neomycin resistance cassette was deleted by Cre recombinase in the germline of *Pax5*^∆OP-Neo/+^ *Meox2*^Cre/+^ mice.

The *Pax5*^∆HD^, *Pax5*^∆OP,HD^, *Pax5*^∆CRD1^, *Pax5*^∆CRD2^, and *Pax5*^∆CRD1,2^ alleles were generated by CRISPR/Cas9-mediated genome editing in mouse zygotes (Yang et al., 2013; Figs. S1 A and S3 A). For this, mouse zygotes (C57BL/6 × CBA) were injected with Cas9 mRNA, a single-guide (sg) RNA targeting the sequence to be mutated (linked to the scaffold tracrRNA), and a single-stranded DNA repair template of 200 nucleotides (Table S6). The *Pax5*^∆HD^ allele was generated by cleavage with sgRNA-1 and sgRNA-2 and homologous recombination of the respective exon 6 repair template (Table S6). The *Pax5*^∆OP,HD^ allele was created by sgRNA-mediated deletion of the HD in *Pax5*^∆OP/∆OP^ zygotes as described above. The *Pax5*^∆CRD1^ allele was generated by deletion of genomic sequences from *Pax5* exon 8 to exon 9 by CRISPR/Cas9-mediated DNA cleavage with sgRNA-3 and sgRNA-4 targeting intronic sequences located upstream of exon 8 and downstream of exon 9, respectively (Fig. S3 A and Table S6). The *Pax5*^∆CRD2^ and *Pax5*^∆CRD1,2^ alleles were created by deletion of the coding sequences at the 5′ end of exon 10 (Fig. 3 A) in the *Pax5*^+^ or *Pax5*^∆CRD1^ allele, respectively, by using sgRNA-5 and the respective exon 10 repair template (Table S6). Mice carrying the different Pax5 mutant allele were genotyped by PCR amplification with the primers shown in Table S6. The PCR product amplified from *Pax5*^∆CRD2/+^ mice was digested with HindIII (Fig. S3 A), resulting in smaller cleaved DNA fragments indicative of the *Pax5*^∆CRD2^ allele. All introduced Pax5 mutations were verified by DNA sequencing of the respective PCR fragment (Table S6). The mutant *Pax5* alleles were backcrossed to the C57BL/6 background for 10 generations prior to analysis.

### Antibodies

The following monoclonal antibodies were used for flow-cytometric analysis of mouse lymphoid organs from 3–12-wk-old mice: B220/CD45R (RA3-6B2), CD2 (RM2-5), CD3 (17A2), CD4 (GK1.5), CD5 (53-7.3), CD8a (53-67), CD11b/Mac1 (M1/70), CD19 (6D5) or CD19 (1D3), CD21/CD35 (7G6), CD23 (B3B4), CD25 (PC61.5), CD44 (IM7), CD49b (HMa2), CD93 (AA4.1), CD117/Kit (ACK2), Gata3 (TWAJ), Gr1 (RB6-8C5), IgD (11-26C), IgM (II/41) or IgM (eb121-15F9), Ly6C (HK1.4), Ly6D (49H4), NK1.1 (PK136), TCRβ (H57-597), Ter119 (Ter-119), and Thy1.2 (53-2.1) antibodies. The anti-Pax5 antibody (1H9; BD Bioscience), which is directed against the amino acids 154–284, was used for intracellular staining of the C-terminal Pax5 mutants, and the anti-Pax5 antibody (D19F8; Cell Signaling), which is directed against the human amino acids surrounding Gln350, was used for intracellular staining of the central Pax5 domain mutants. The polyclonal anti-Pax5 antibody, which is directed against amino acids 17–145 (Adams et al., 1992), was used for ChIP and Co-IP experiments. The anti-Brg1 antibody (ab110641; Abcam) was used for Brg1 ChIP analysis.

### Definition of cell types by flow cytometry

The different hematopoietic cell types of the mouse were identified by flow cytometry as follows: LMPP (CD19^−^Lin^−^CD135^+^Kit^+^Sca1^+^CD127^−^), ALP (Lin^−^CD135^+^CD127^+^Ly6D^−^), BLP (Lin^−^CD135^+^CD127^+^Ly6D^+^), Pax5-deficient B cell progenitors (Lin^−^Ly6D^+^Kit^hi^B220^+^), pro-B (B220^+^CD19^+^Kit^+^CD2^−^IgM^−^IgD^−^) or (B220^+^CD19^+^Kit^+^CD25^−^IgM^−^IgD^−^), pre-B (B220^+^CD19^+^Kit^−^CD2^+^IgM^−^IgD^−^) or (B220^+^CD19^+^Kit^−^CD25^+^IgM^−^IgD^−^), immature B (B220^+^CD19^+^IgM^hi^IgD^−^), recirculating B (B220^+^CD19^+^IgM^+^IgD^hi^), MZ B (B220^+^CD19^+^CD93^−^CD21^hi^CD23^lo/−^), FO B (B220^+^CD19^+^CD93^−^CD21^int^CD23^hi^), total B cells (B220^+^CD19^+^), ETP/DN1 (CD19^−^Lin^−^CD25^−^CD44^+^Kit^hi^), DN2a (CD19^−^Lin^−^CD25^+^CD44^+^Kit^hi^), DN2b (CD19^−^Lin^−^CD25^+^CD44^+^Kit^int^), DN3a (Lin^−^Thy1.2^+^CD25^hi^CD44^−^CD28^−^CD71^−^), and DN3b (Lin^−^Thy1.2^+^CD25^hi^CD44^−^CD28^+^CD71^+^). LMPP, ALP, BLP, and the Pax5-deficient B cell progenitors were defined by gating away Lin^+^ cells with a cocktail of anti-CD3, CD4, CD8a, CD11b, CD49b, Gr1, Ly6C, NK1.1, TCRβ, and Ter-119 antibodies. A second antibody cocktail (Lin^+^) consisting of anti-CD4, CD8a, CD11b, TCRβ, TCRγδ, CD11c, NK1.1, Gr1, and Ter-119 antibodies was used for flow-cytometric sorting of the T cell precursors DN1, DN2a, DN2b, DN3a, and DN3b. Flow-cytometric analysis and sorting were performed on LSRFortessa (BD Biosciences) and FACSAria III (BD Biosciences) machines, respectively. FlowJo Software (Treestar) was used for data analysis.

### Intracellular staining for flow cytometry

Intracellular Pax5 staining of pro-B cells (Fig. 4, A and B; and Fig. S1 C; and Fig. 8, C and D) was performed after fixation–permeabilization with the Foxp3 Staining Buffer Set (eBioscience) by staining with the anti-Pax5 antibody (1H9; BD Bioscience) or anti-Pax5 antibody (D19F8; Cell Signaling).

### In vitro T cell differentiation

The experiment was performed as described (Höflinger et al., 2004). In short, ex vivo sorted pro-B cells were cultured on OP9 cells in IL-7–containing Iscove’s modified Dulbecco’s medium (IMDM; Nutt et al., 1997) for 4 d. Half of the pro-B cells were then transferred to OP9-DL1 cells (Schmitt and Zúñiga-Pflücker, 2002) in α-MEM medium containing 10% FCS, 50 μM β-mercaptoethanol, 2 mM glutamine, 1 mM sodium pyruvate, IL-7, and Flt3L (Höflinger et al., 2004). On day 7 after transfer onto OP9-DL1, the lymphoid cells on the OP9-DL1 and OP9 feeder cells were harvested and analyzed by flow cytometry.

### ChIP-seq experiments

Pro-B cells were short-term cultured on OP9 cells in IL-7–containing IMDM (Nutt et al., 1997) followed by crosslinking with 1% formaldehyde (Sigma-Aldrich) for 10 min. Nuclei were prepared and lysed in the presence of 0.25% SDS, followed by sonication of the chromatin with the Bioruptor Standard (Diagenode). IP was performed with an anti-Pax5 paired domain antibody (Adams et al., 1992), and the precipitated DNA (1–2 ng) was used for library preparation and subsequent Illumina deep sequencing (Table S7). For Brg1 ChIP analysis, in vitro cultured pro-B cells were sequentially crosslinked at room temperature with 1.5 mM ethylene glycol bis(succinimidylsuccinate) for 15 min and then with 1% formaldehyde for 10 min, followed by ChIP analysis with an anti-Brg1 antibody (ab110641; Abcam), as described (Wang et al., 2020).

### cDNA preparation for RNA-seq

RNA from ex vivo sorted pro-B cells was isolated with a RNeasy Plus Mini kit (Qiagen), and mRNA was obtained by poly(A) selection with a Dynabeads mRNA purification kit (Invitrogen) followed by cDNA synthesis as described (Calderón et al., 2021).

### Library preparation and Illumina deep sequencing

About 1–5 ng of cDNA or ChIP-precipitated DNA were used for generating sequencing libraries with the NEBNext Ultra Ligation Module and NEBNext End Repair/dA-tailing module, as described (Calderón et al., 2021). Cluster generation and sequencing were carried out by using the Illumina HiSeq 2000 system with a read length of 50 nucleotides (Table S7).

### MS analysis of Co-IP proteins

The nuclear extracts were prepared from in vitro cultured pro-B cells (1–2 × 10^8^) as described (McManus et al., 2011). IP was performed with an anti-Pax5 paired domain antibody (Adams et al., 1992). For this, 10 ml Protein A Mag Sepahrose Xtra beads (GE Healthcare) per 1 mg nuclear extract were precleared by incubation with PBS containing 1 mg/ml bovine serum albumin for 2 h at 4°C. Subsequently, the nuclear extract was incubated with the beads for 2 h at 4°C with rotation, followed by washing five times with ice-cold wash buffer containing detergents (20 mM Tris, pH 8.0, 1.5 mM MgCl_2_, 10% glycerol, 250 mM NaCl, 0.15% NP-40) and seven times with ice-cold wash buffer without detergents (20 mM Tris-HCl, pH 7.5, 150 mM NaCl). Pelleted beads were stored at −20°C until further processing for MS.

Proteins were digested off the beads using Lys-C followed by reduction, alkylation, and a subsequent digestion with trypsin as described (de Almeida et al., 2021). A similar aliquot of each sample (10%) was analyzed by nanoLC-MS/MS using an UltiMate 3000 RSLC nano system operating a PepMap trap and analytical column and being coupled to an Exploris 480 mass spectrometer equipped with a Nanospray Flex ion source (all parts from Thermo Fisher Scientific). HPLC and MS were operated as described (de Almeida et al., 2021). For peptide identification, the RAW files were searched using MS Amanda version 2.0.0.16129 (Dorfer et al., 2014) in the framework of Proteome Discoverer (version 2.5.0.400; Thermo Fisher Scientific) against the UniProt mouse reference database (21,961 sequences; 11,726,931 residues), supplemented with common contaminants and the sequence of Pax5, using the following search parameters: peptide and fragment mass tolerance ±10 parts per million, number of missed tryptic cleavages ≤2, Cys b-methylthiolation as a fixed modification, as well as Met oxidation and other common posttranslational modifications as variable modifications. Identifications were filtered to 1% false discovery rate on protein and posttranslational modification level using the Percolator algorithm integrated into Proteome Discoverer, and on top, an MSAmanda score cutoff of ≥150 was applied.

Peptides were subjected to label-free quantification using IMP-apQuant (Doblmann et al., 2019). Proteins were quantified by summing unique and razor peptides and applying intensity-based absolute quantification (Schwanhäusser et al., 2011). Proteins were filtered to be identified by a minimum of three quantified peptides. Protein abundance was normalized to equal amounts of the bait (Pax5) per condition. The statistical significance of differentially abundant proteins was determined using limma (Smyth, 2004). Mitochondrial proteins were considered as contaminants and excluded from further analysis.

### Bioinformatic analysis of RNA-seq data

All sequence reads of the different samples that passed the Illumina quality filtering were considered for adapter trimming and subsequent alignment to the mouse genome assembly version of December 2011 (GRCm38/mm10) using STAR 2.4.2a (Dobin et al., 2013) in the transcriptome-guided alignment mode. All reads were trimmed to 50 base pairs to obtain comparable alignments. Read counts per gene were obtained with Rsubread 2.8.0 (Liao et al., 2014). The datasets were grouped according to the genotype and were analyzed using R 4.1.2 9 (https://www.r-project.org), Bioconductor 3.14 (Huber et al., 2015), and the R package DESeq2 version 1.34 (Love et al., 2014). Genes with low expression (counts per million < 1 in all samples) were removed from the analysis. Batch effects were corrected with limma 3.50.0 (Ritchie et al., 2015). The normalizations and dispersion estimations of the samples were conducted using the default DESeq2 settings. Variance-stabilizing transformations were computed with the blind option set to “False.” Variance-stabilized counts were transformed from the log_2_ to the log_10_ scale for generating scatterplots. The default DESeq2 pairwise setup (model design formula ∼genotype; Wald test) was used for comparisons between different conditions. If not stated otherwise in the text, genes with an adjusted P value of <0.05, an absolute fold-change of >3, and a mean TPM value (averaged within conditions) of >5 in one of the conditions (genotypes) were called as significantly differentially expressed. Immunoglobulin and histone genes were filtered from the final list of significantly differentially expressed genes. Genes were manually annotated based on the literature and Ingenuity pathway analysis (Krämer et al., 2014). All heatmaps were drawn with ComplexHeatmap 2.10 (Gu et al., 2016). Sequence reads obtained by RNA-seq analysis of LMPP, DN1, DN2a, DN2b, DN3a, and DN3b cells were aligned with TopHat 1.4.1 (Trapnell et al., 2009). TPMs were calculated as described (Wagner et al., 2012). The database generation of the RefSeq-annotated genes was performed as previously described (Wöhner et al., 2016), based on a RefSeq data download from December 6, 2018.

### Bioinformatic analysis of ChIP-seq and ATAC-seq data

All sequence reads of the different samples that passed the Illumina quality filtering were considered for adapter trimming and subsequent alignment to the mouse genome assembly version of December 2011 (GRCm38/mm10) using bowtie 1.0.0 (Langmead et al., 2009). Replicates were concatenated and peaks were called using MACS 2.2.5 (Zhang et al., 2008), considering a Rag2^−/−^ pro-B cell input sample (GSM1145867, GSM1296537) as a control in case of the Pax5 ChIP-seq analysis and the merged input replicates (231740, 231741, 231742) as a control in case of the Brg1 ChIP-seq analysis. Peaks were subsequently filtered for P value of <10^−10^ and submitted to irreproducible discovery rate (IDR) thresholding by applying Idr 2.0.4 (Li et al., 2011) in oracle mode. To obtain a comprehensive list of all interesting ChIP-seq and/or ATAC-seq peaks, the summits of ChIP-seq and ATAC-seq peaks were grouped into clusters and subsequently filtered by overlapping them with IDR-filtered regions to obtain only relevant clusters using MULTOVL 1.2 (Aszódi, 2012). To increase the resolution within peaks, clusters with summits that were present at a distance of >150 bp apart were separately analyzed. Reads over the resulting subpeaks were counted using deepTools 3.3.1 multiBamSummary (Ramírez et al., 2014) by extending the reads to 200 bp (ChIP-seq) or considering the entire paired-end-sequenced DNA fragments (ATAC-seq) and were transformed to reads per millions (RPM). All sites were assigned to genes as described (Revilla-i-Domingo et al., 2012), based on the RefSeq database that was processed as described below. In particular, peaks overlapping with the promoter region (−2.5 to +1 kb relative to the transcription start site [TSS]) were termed “TSS” peaks and peaks present in the gene body (+1 kb from the TSS to the transcription end site) were referred to as intragenic enhancer peaks. We next assumed that the peak with the greatest ChIP and/or ATAC signal change between pro-B cells of the different genotypes analyzed is the most interesting peak with regard to gene regulation. As for each gene, several peaks can be assigned to the promoter region or gene body, the peak with the maximum RPM difference in the mutant versus wild-type condition was finally selected and plotted per gene. Read coverages were calculated with the BEDTool program version 2.27.1 (Quinlan and Hall, 2010) and were normalized to RPM using the SAMTools version 1.9 (Li et al., 2009) as well as the KentTools version 20190507 (Kuhn et al., 2013). Displayed read coverage tracks are based on UCSC genome browser screenshots (Kent et al., 2002). We used DESeq2 version 1.34 (Love et al., 2014) to normalize the ATAC-seq data and to calculate the data of the principal component analysis.

### Statistical analysis

Statistical analysis was performed with the GraphPad Prism 7 software. Two-tailed unpaired Student’s *t* test analysis was used to assess the statistical significance of one observed parameter between two experimental groups. One-way ANOVA was used when more than two experimental groups were compared followed by Dunnett’s multiple comparison test.

### Online supplemental material

Fig. S1 describes the generation and characterization of the *Pax5*^∆OP^, *Pax5*^∆HD^, and *Pax5*^∆OP,HD^ alleles. Fig. S2 displays the heatmap of differentially expressed genes in *Pax5*^∆OP/∆OP^, *Pax5*^∆HD/∆HD^, or *Pax5*^∆OP,HD/∆OP,HD^ pro-B cells compared with *Pax5*^+/+^ pro-B cells. Fig. S3 describes the generation and characterization of the *Pax5*^∆CRD1^, *Pax5*^∆CRD2^, and *Pax5*^∆CRD1,2^ alleles. Fig. S4 shows the differential dependency of Pax5-regulated genes on the function of the C-terminal domains. Fig. S5 displays the expression profiles of Pax5-regulated genes with a potential role in B-lineage commitment. Table S1 contains the mRNA-seq data of all Pax5-regulated genes that depend on the central domains. Table S2 contains the mRNA-seq data of all Pax5-regulated genes that require the C-terminal domains for their expression. Table S3 contains the mRNA-seq data of the genes that are differentially expressed between *Pax5*^Prd/∆^ and *Pax5*^∆/∆^ progenitors. Table S4 contains the MS data used for the identification of proteins that interact with the C-terminal domains of Pax5. Table S5 contains the mRNA-seq data used for the identification of Pax5-regulated genes that are potentially involved in B-lineage commitment (Fig. 8 F). Table S6 contains the oligonucleotide sequences used for PCR analysis or CRISPR-Cas9 mutagenesis. Table S7 describes all Illumina sequencing experiments generated for this study.

## Supplementary Material

Table S1 contains the mRNA-seq data of all Pax5-regulated genes that depend on the central domains.

Table S2 contains the mRNA-seq data of all Pax5-regulated genes that require the C-terminal domains for their expression.

Table S3 contains the mRNA-seq data of the genes that are differentially expressed between *Pax5*^Prd/∆^ and *Pax5*^∆/∆^ progenitors.

Table S4 contains the mass-spectrometric data used for the identification of proteins that interact with the C-terminal domains of Pax5.

Table S5 contains the mRNA-seq data used for the identification of Pax5-regulated genes that are potentially involved in B-lineage commitment (Fig. 8 F).

Table S6 contains the oligonucleotide sequences used for PCR analysis or CRISPR-Cas9 mutagenesis.

Table S7 describes all Illumina sequencing experiments generated for this study.

SourceData FS3 is the source file for Fig. S3.

SourceData FS4 is the source file for Fig. S4.

## Data Availability

The RNA-seq, ChIP-seq, and ATAC-seq data reported in this study (Table S7) are available at the Gene Expression Omnibus repository under the accession number GSE224793. The Co-IP–MS data are available at the ProteomeXchange Consortium PRIDE repository under the accession number PXD040424.
