# Supplementary material for: Essential role of the Pax5 C-terminal domain in controlling B cell commitment and development
Source: J Exp Med. 2023 Sep 19;220(12):e20230260. doi: 10.1084/jem.20230260 (PMC10509461; doi:10.1084/jem.20230260)
Supplement: SourceData FS3 — is the source file for Fig. S3. [file JEM_20230260_SourceDataFS3.pdf]

Gruenbacher et al.

Uncropped Western blot

Figure S3B

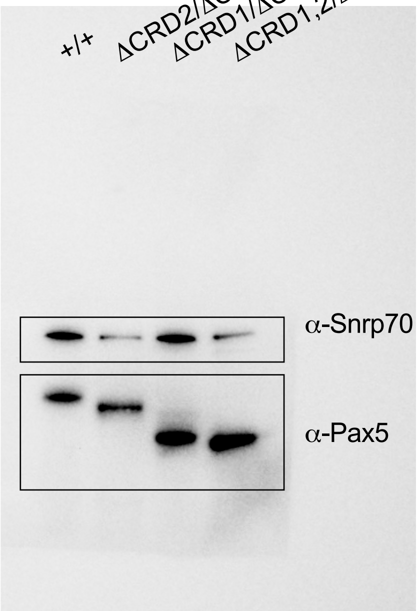

Overlap with colorimetric exposure to visualize ladder

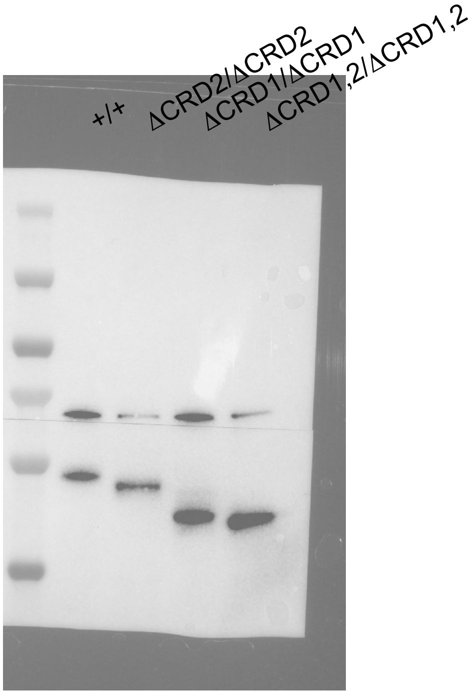

Note:
- The sizes of the marker proteins are indicated in kDa (PageRuler™ Plus Prestained Protein Ladder).
